# *Sirt6* loss activates *Got1* and facilitates cleft palate through abnormal activating glycolysis

**DOI:** 10.1038/s41419-025-07465-8

**Published:** 2025-03-06

**Authors:** Xiaotong Wang, Xige Zhao, Xiaoyu Zheng, Xia Peng, Jing Chen, Yijia Wang, Zhiwei Wang, Mingyue Meng, Juan Du

**Affiliations:** 1 Laboratory of Orofacial Development, Laboratory of Molecular Signaling and Stem Cells Therapy, Molecular Laboratory for Gene Therapy and Tooth Regeneration, Beijing Key Laboratory of Tooth Re-generation and Function Reconstruction, Capital Medical University School of Stomatology, Fanjiacun Road No.9, Beijing, 100070 China; 2 Department of geriatric dentistry, Capital Medical University School of Stomatology, Fanjiacun Road No.9, Beijing, 100070 China

**Keywords:** Embryogenesis, Epigenetics, Bone development

## Abstract

Cleft palate (CP) is a common congenital craniofacial malformation, which is caused by a combination of genetic and environmental factors. However, its underlying mechanism has not been elucidated. Sirtuin6 (*SIRT6*) mutation has been associated with craniofacial anomalies in humans. This study further defined the role of *Sirt6* in palatogenesis by investigating the specific inactivation of *Sirt6* in Wnt1-expressing cell lineages. Here, we demonstrated that *Sirt6* conditioned knockout (*Sirt6* cKO) could inhibit the osteogenesis of the palate which facilitated the occurrence of CP. Specifically, *Sirt6* deficiency promoted the expression of glutamine oxaloacetic transaminase 1 (*Got1*) and glycolysis through deacetylation inhibition, which increased the proliferation of mouse embryonic palatal mesenchyme (MEPM) cells through the GOT1-lactate dehydrogenase A (LDHA)-transforming growth factor beta receptor 1 (TGFBR1) pathway in the early stage and inhibited the osteogenic differentiation of MEPM cells through the GOT1-LDHA-bone morphogenetic protein 2 (BMP2) pathway in the late stage. Notably, if there was a disturbance of the environment, such as retinoic acid (RA), the occurrence of CP increased. Also, the enhanced acetylation of histone 3 lysine 9 (H3K9) in *Got1* induced by *Sirt6* deficiency was mediated by the acetylase tat-interacting protein 60 (TIP60) rather than acetyltransferase p300 (P300). Additionally, inhibition of *Got1* partially saved the promoting effect of *Sirt6* cKO on the CP. Our study reveals the role of *Sirt6* in facilitating CP, with *Got1* as the primary driver.

## Introduction

Cleft palate (CP) is a common congenital craniofacial malformation and is now generally believed to be caused by a combination of genetic and environmental factors [1]. CP not only causes facial abnormalities, but also affects patients’ eating, hearing, and mental health [2–4]. Secondary palate development is an essential morphogenetic event of the midface when craniofacial development is derived from the migration of cranial neural crest cells (CNCCs) from the mid-hindbrain to the anterior [5]. Palatogenesis involves the vertical descent, horizontal elevation, fusion, and ossification of the palatal shelves, any disturbance can cause abnormal palate development including CP [1, 6]. Therefore, further exploration of the biological processes of palatogenesis may help promote potential prevention and prenatal interceptive therapy of CP.

Recently, the regulation of metabolism in embryonic development has been focused [7, 8]. For instance, the metabolic state of embryonic stem cells (ESCs) is enhanced at glycolysis when the ESCs proliferate and maintain pluripotency, and is weakened as the ESCs start to differentiate into specific derivatives [9, 10]. Notably, the glycolytic activity is enhanced before gastrulation as it is needed to help properly execute the development of gastrulation in *Xenopus* [11]. Andrographolide inhibits murine embryonic neuronal development through a glycolytic pathway [12]. Our previous study also revealed there was glucose reprogramming from glycolytic to oxidative phosphorylation (OXPHOS) during palate development [13], but the specific mechanisms that affected the glucose reprogramming have not been clarified.

The histone deacetylase Sirtuin6 (*Sirt6*) is a member of the Sirtuin family of nicotinamide adenine dinucleotide (NAD^+^)-dependent enzymes, and implicated in aging, metabolic, inflammation, and cardiovascular diseases [14–17]. Early study has reported that four fetuses identified a homozygous *SIRT6* mutation reveal a number of prenatal abnormalities, including intrauterine growth restriction (IUGR), microcephaly, craniofacial anomalies, congenital heart defects, and sex reversal in male fetuses. Cephalic and craniofacial fetal abnormalities include cerebellar hypoplasia, decreased head circumference, and frontal bossing [18]. *Sirt6*-deficient mice present with reduced body size, loss of subcutaneous fat, lordokyphosis, osteopenia, and lethal hypoglycemia at postnatal 2–3 weeks, highlighting the regulatory role played by *Sirt6* in glucose metabolism [19]. Further, SIRT6 promotes mitochondrial biogenesis and mitophagy during doxorubicin treatment, thereby remodeling from glycolysis to mitochondrial respiration, thus protecting cardiomyocytes against the energy deficiency induced by doxorubicin [20]. In addition, it has been showed that SIRT6 regulates telomeric chromatin and expression of downstream genes through its histone 3 lysine 9 (H3K9), H3K18, and H3K56 deacetylase activity [21–23]. However, the precise molecular mechanism of *Sirt6* in palate development remains unclear, and whether *Sirt6* is involved in glucose metabolism in palatogenesis.

In this research, we aimed to highlight the role of *Sirt6* in palate development and explore the underlying molecular mechanism. Wnt1-Cre has been widely used for specific gene knockouts in CNCC lineages [24]. Therefore, we conditionally inactivated the *Sirt6* gene in CNCC lineages using the Cre/loxp recombination system. Following this, we investigated how *Sirt6* affected palate development through Glutamate oxaloacetate transaminase 1 (*Got1*) and glycolysis, which provided a novel potential therapeutic target for CP.

## Materials and methods

Materials and methods are listed in Supplementary materials and methods.

## Results

### *Sirt6* cKO affects the development of the palate in mice and aggravates the formation of CP

**Fig. 1:**
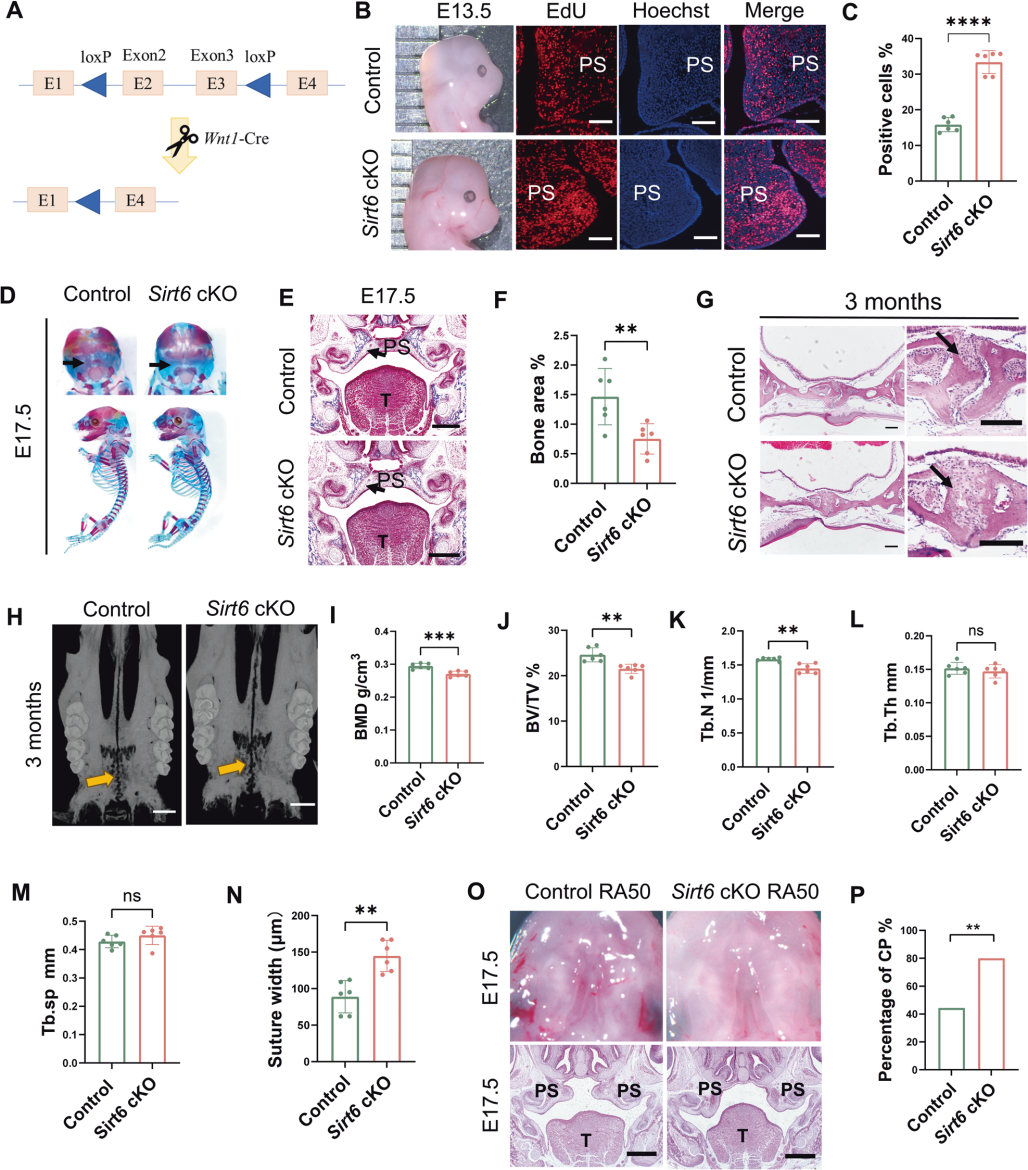
Wnt1-cre-mediated conditional loss of *Sirt6* affects palatogenesis in mice and causes an increased propensity for malformation of the palate. **A** Schematic of genomic DNA region around exon 2 and exon 3 of *Sirt6* in mice. **B**, **C** The appearance of the skull at E13.5 and EdU staining of E13.5 palatal tissues, Bar: 50 μm, PS palatal shelves, *n* = 6. **D** Alizarin red and Alcian blue staining skeletons of E17.5 Control and *Sirt6* cKO mice. Note ossification of palatine bone in Control and *Sirt6* cKO mice (black arrows). **E**, **F** Masson trichrome staining of the maxillofacial region, Bar: 200 μm, *n* = 6. Note ossification of palate bone in Control and *Sirt6* cKO mice (black arrows), PS palatal shelves, T tongue. **G** Haematoxylin & Eosin staining of histological sections of 12 weeks palate samples to observe the morphological change of the palate. Bar: 100 μm, *n* = 6. **H** Three-dimensional micro-CT reconstructions of palate from Control and *Sirt6* cKO mice at 3 months by Ctvox, Bar: 1 mm. Note median palatine suture in Control and *Sirt6* cKO mice (orange arrows). **I**–**M** Micro-CT analysis of bone changes (BMD, BV/TV, Tb.N, Tb.Th, and Tb.Sp) on the palate of Control and *Sirt6* cKO mice at 12 weeks, *n* = 6. **N** Quantitative results of palatal median suture width, *n* = 6. **O** Macroscopic appearance of palates at E17.5 and Haematoxylin & Eosin staining of histological sections of E17.5 skull samples, RA was used at 50 mg/kg, Bar: 200 μm. **P** Quantitative analysis of cleft palate rate, *n* = 7 pregnant mice, **p < 0.01, ***p < 0.001, ****p < 0.0001, ns not significant.

To investigate the expression pattern of SIRT6 at different stages of mouse palate development, we evaluated SIRT6 levels in palatal tissue of different stages. Our data demonstrated that *Sirt6* gradually decreased from embryo (E) 12.5 to 18.5 at both mRNA and protein levels (Supplementary Fig. 1A–C). And most of SIRT6 is expressed in the mesenchyme of the palatal shelves (Supplementary Fig. 1D, E). The special spatiotemporal expression of SIRT6 suggested that SIRT6 might play an essential role in palatogenesis. We then built *Sirt6* conditional knockout (Wnt1-Cre/*Sirt6*^*loxp/loxp*^, *Sirt6* cKO) mice to explore its effect on CP etiology (Fig. 1A). We performed genotyping and protein expression of SIRT6 in palate to confirm the knockout in the CNCCs (Supplementary Fig. 1F–L). The majority of *Sirt6* cKO mice were born full-term and lived to maturity without visible craniofacial malformations. Male mice of *Sirt6* cKO were fertile, and female mice of *Sirt6* cKO were infertile. Considering that the palate grows rapidly in size due to high rates of cellular proliferation at E13.5, and the palatine calcification occurs at E17.5 [25, 26], the phenotypes of *Sirt6* cKO mice at different palatal developmental stages were observed. Firstly, compared with the Control group, the heads of the *Sirt6* cKO mice were bigger, and the proliferative activity of the palatal process was enhanced at E13.5 *Sirt6* cKO fetuses (Fig. 1B, C). In contrast, while there was no abnormal skeletal patterning compared with the Control littermates, lower calcification of the palatine bone was observed in *Sirt6* cKO mice at E17.5 (Fig. 1D–F). To confirm the result, we subsequently investigated the palatine bone of the adult mice. It was displayed that the trabecular structure of the midpalate bone became blurred in *Sirt6* cKO mice when compared with the Control at 12 weeks by HE (Fig. 1G). Furthermore, *Sirt6* cKO mice exhibited significant decreases in bone mineral density (BMD), trabecular number (Tb.N), and tibial subchondral bone volume fraction (BV/TV), without changes in trabecular separation (Tb.Sp) and trabecular thickness (Tb.Th) under micro-CT (Fig. 1H–M), indicating impaired bone sclerosis in this group. In addition, the widths of a median palatine suture in *Sirt6* cKO were increased compared to Control mice (Fig. 1N). These results indicated that *Sirt6* played a dual role in palate development, as *Sirt6* cKO promoted proliferation at E13.5 and suppressed osteogenesis at later E17.5 and postnatal 12 weeks.

As no significant CP was observed in *Sirt6* cKO mice, we hypothesized that this might be due to the dual role of SIRT6 in palatogenesis, so the superposition of the two effects masked the phenotype. Therefore, we used 50 mg/kg retinoic acid (RA), the metabolite of vitamin A and a common chemical inducing CP, to investigate whether the interaction of *Sirt6* cKO with environmental factors influenced the occurrence of CP. Interestingly, the CP was observed after *Sirt6* cKO combined with RA, and the rate of CP in the *Sirt6* cKO group (80%, total 7 pregnant mice with 20 *Sirt6* cKO fetuses collected, 16 with CP) was significantly higher than that with RA only (44.4%, total 7 pregnant mice with 27 Control fetuses collected, 12 with CP) (Fig. 1O, P and Supplementary Table 1). Similar results were observed in a combination of SIRT6 inhibitor (OSS_128167) and RA (Supplementary Fig. 1M, N and Supplementary Table 2). These results confirmed that *Sirt6* deficiency increased the genetic susceptibility to CP. When combined with environmental factors, SIRT6 deficiency increased the occurrence of CP.

### *Sirt6* deficiency promotes the proliferation of MEPM cells and represses their osteogenic differentiation in vitro

**Fig. 2:**
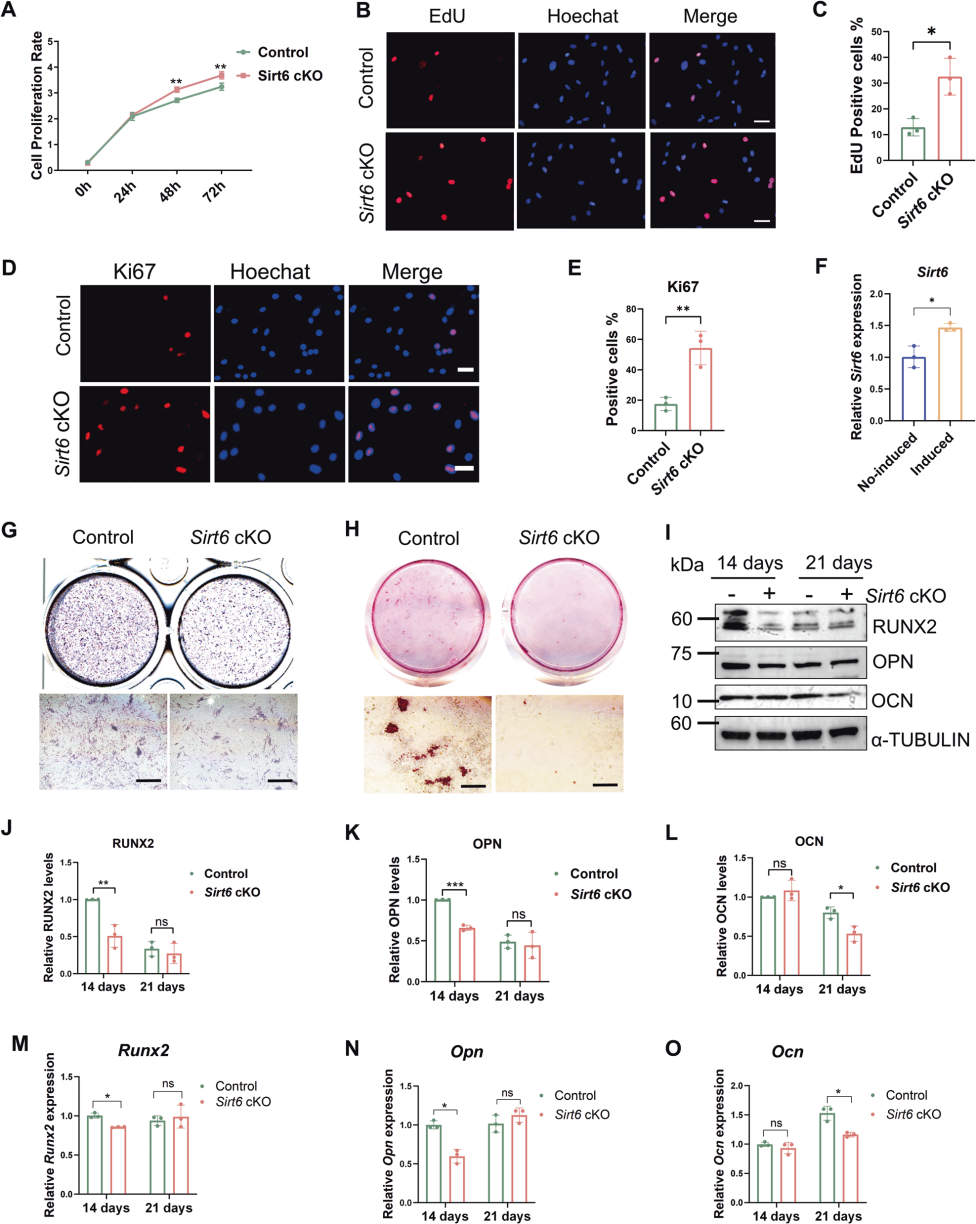
*Sirt6* deficiency promotes the proliferation of MEPM cells and reduces their osteogenic capacity in vitro. **A** CCK-8 was used to detect the effect of *Sirt6* deficiency on cell proliferation between Control and *Sirt6* loss MEPM cells in E13.5, *n* = 5. **B**, **C** E13.5 MEPM cell proliferation was measured by an EdU staining assay, Bar: 50 μm, *n* = 3. **D**, **E** Immunofluorescence analysis of Ki67 in E13.5 MEPM cells, Bar: 30 μm, *n* = 3. **F** The expression of SIRT6 before and after osteogenesis was analyzed by qRT-PCR, *n* = 3. **G**, **H** ALP (**G**) and Alizarin red (**H**) staining was used to observe the changes of osteogenic differentiation of Control and *Sirt6* loss MEPM cells in E15.5, Bar: 200 μm. **I**–**L** After 14 and 21 days of osteogenic induction, WB was used to analyze the changes of RNUX2 (**J**), OPN (**K**), and OCN (**L**) in the Control and *Sirt6* loss MEPM cells, *n* = 3. **M**–**O** After 14 and 21 days of osteogenic induction, qRT-PCR was used to analyze the changes of *Runx2* (**M**), *Opn* (**N**), and *Ocn* (**O**) in the Control and *Sirt6* loss MEPM cells, *n* = 3. *p < 0.05, **p < 0.01, ns not significant.

To assess the effect of *Sirt6* on mouse embryonic palatal mesenchyme (MEPM) cells, and to get closer to the physiological state in vivo, we isolated E13.5 and E15.5 primary MEPM cells from *Sirt6* cKO mice and Control littermates, respectively. The proliferative activity of E13.5 *Sirt6* deficiency MEPM cells was significantly higher than the Control (Fig. 2A–E). Conversely, *Sirt6* expression increased after osteogenic induction in E15.5 MEPM cells (Fig. 2F). And, E15.5 *Sirt6* deficiency MEPM cells displayed a significant reduction of osteogenic markers osteopontin (OPN) and runt-related transcription factor 2 (RUNX2) in 14 days, and decreased expression of osteocalcin (OCN) in 21 days (Fig. 2I–O) than Control MEPM cells at the protein and mRNA levels. Furthermore, as a SIRT6 agonist, MDL-800 inhibited the proliferative activity of E13.5 MEPM cells and promoted the osteogenic differentiation of E15.5 MEPM cells (Supplementary Fig. 2), which confirmed the effects of *Sirt6* loss on MEPM cells. The above results indicated that *Sirt6* deficiency promoted proliferative activity and repressed osteogenic differentiation in MEPM cells at different stages in vitro.

### *Sirt6* modulates the proliferation and osteogenesis of the palate through TGFBR1 and BMP2, respectively

**Fig. 3:**
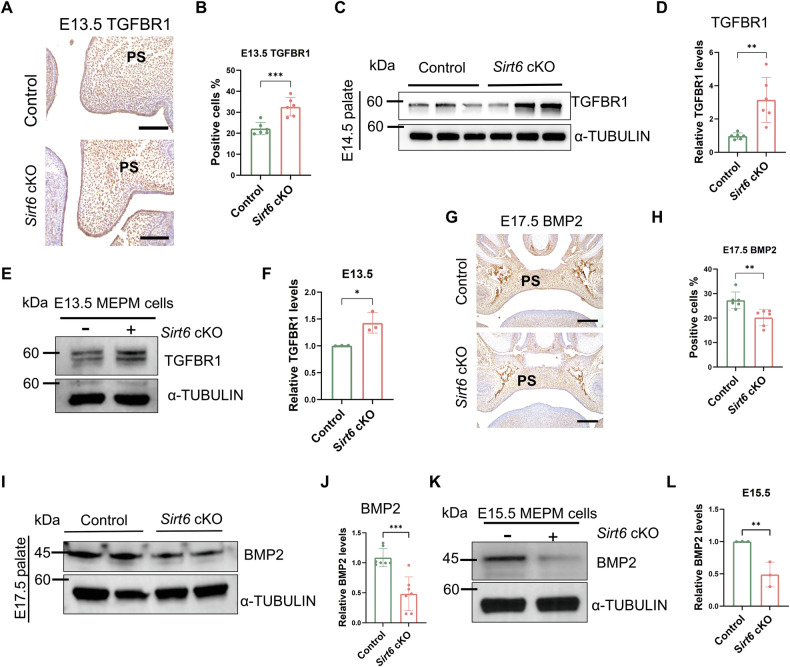
*Sirt6* modulates the palate development through TGFBR1 and BMP2. **A**, **B** Representative immunohistochemistry of TGFBR1 in the palate of the E13.5 Control and *Sirt6* cKO mice and quantitative analysis, Bar: 50 μm, *n* = 6. **C**, **D** Western blot analysis of protein expression in palatal tissues from E14.5 Control and *Sirt6* cKO mice, *n* = 6. **E**, **F** Western blot analysis of protein expression in E13.5 MEPM cells, *n* = 3. **G**, **H** Representative immunohistochemistry of BMP2 in the palate of the E17.5 Control and *Sirt6* cKO mice and quantitative analysis, Bar: 100 μm, *n* = 6. **I**, **J** Western blot analysis of protein expression in palatal tissues from E17.5 Control and *Sirt6* cKO mice, *n* = 6. **K**, **L** Western blot analysis of protein expression in E15.5 MEPM cells, *n* = 3. *p < 0.05, **p < 0.01, ***p < 0.001.

Transforming growth factor beta (TGF-β) signaling mediates a wide range of biological activities in development and diseases [27]. And TGF-β signaling plays an essential role in the proliferation of MEPM cells [28]. Therefore, we evaluated transforming growth factor beta receptor 1 (TGFBR1) expression and observed that *Sirt6* loss-activated TGFBR1 expression in E13.5 and E14.5 palate tissues (Fig. 3A–D), and E13.5 MEPM cells (Fig. 3E, F), suggesting that *Sirt6* loss might affect TGFBR1 and thus affect early palatal process proliferation.

Bone morphogenetic protein 2 (BMP2) plays a key regulatory role in bone metabolism [29, 30], and it is indicated that haploinsufficiency of *BMP2* plays a crucial role in the formation of CP [31]. It was also observed that *Sirt6* loss inhibited the expression of BMP2 in E17.5 palate tissues (Fig. 3G–J) and E15.5 MEPM cells (Fig. 3K, L), so *Sirt6* loss might affect the formation of palatine bone through BMP2 at the late stage.

### Glucose metabolism reprogramming of the palate is disturbed when *Sirt6* is inhibited

**Fig. 4:**
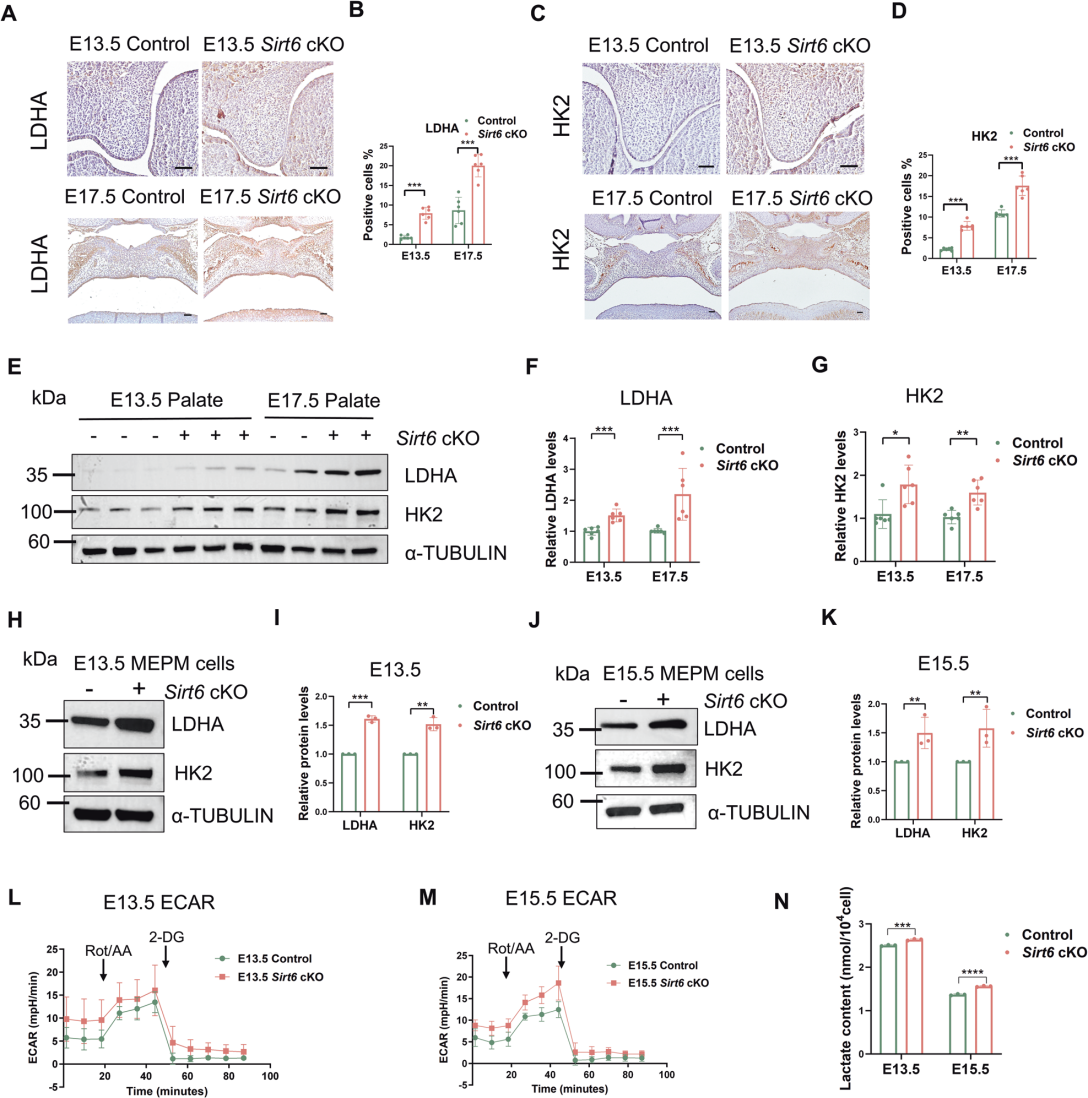
*Sirt6* loss disturbs glucose metabolism reprogramming of the palate. **A**–**D** Representative immunohistochemistry of LDHA (**A, B**) and HK2 (**C**, **D**) in the palate of the Control and *Sirt6* cKO mice and quantitative analysis, Bar: 50 μm, *n* = 6. **E**–**G** Western blot analysis of LDHA and HK2 expression in E13.5 and E17.5 palatal tissues from control and *Sirt6* cKO mice. α-TUBULIN was used as a loading control. Quantification of protein bands, *n* = 6. **H**, **I** Western blot analysis of LDHA and HK2 expression in E13.5 MEPM cells, *n* = 3. **J**, **K** Western blot analysis of LDHA and HK2 expression in E15.5 MEPM cells, *n* = 3. **L**, **M** The dynamic changes of glycolysis in E13.5 (**L**) and E15.5 (**M**) MEPM cells were measured by ECAR using a Seahorse extracellular flux analyzer, *n* = 3. **N** Quantitative analysis of lactate was performed by lactate content detection kit, *n* = 3. *p < 0.05, **p < 0.01, ***p < 0.001.

Metabolism reprogramming is important for embryonic development [8], and our previous studies found that there existed glucose metabolism reprogramming during palatogenesis [13]. We then checked the changes of glycolysis in *Sirt6* loss MEPM cells. Lactate dehydrogenase A (LDHA) and hexokinase 2 (HK2) are key components of the glycolysis pathway [32, 33]. Our results revealed that LDHA and HK2 levels were increased in the *Sirt6* cKO palatal tissues both at E13.5 and E17.5 by immunohistochemical staining and western blot (Fig. 4A–G). Similarly, the same results were observed in 13.5 and 15.5 MEPM cells (Fig. 4H–K), which showed that glycolysis was continuously up-regulated in the palate of *Sirt6* cKO mice. Next, *Sirt6* deficiency was detected to activate glycolysis of E13.5 and E15.5 MEPM cells by ECAR (Fig. 4L, M). Furthermore, the concentrations of lactate in E13.5 and E15.5 MEPM cells of *Sirt6* deficiency were higher than those in the Control (Fig. 4N). All these meant a disturbed glucose metabolism reprogramming happened during palate development which might relate to the occurrence of CP.

### Loss of *Sirt6* facilitates H3K9 acetylation and increases *Got1* expression

**Fig. 5:**
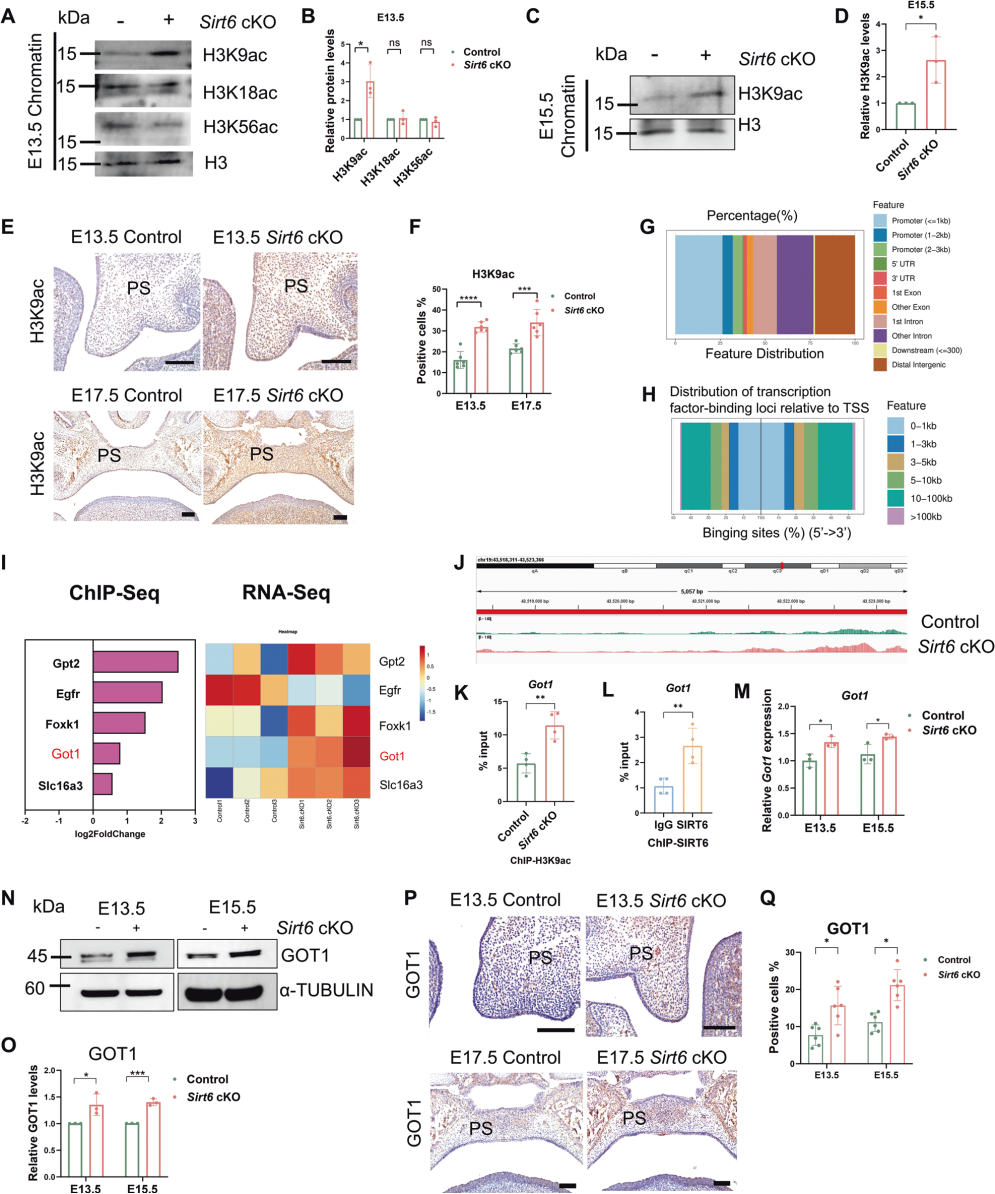
Loss of *Sirt6* facilitates H3K9 acetylation and increases *Got1* expression. **A**–**D** The levels of H3K9ac, H3K18ac, and H3K56ac were measured by WB, *n* = 3. **E**, **F** Immunohistochemical analysis against H3K9ac, Bar: 50 μm, *n* = 6. **G**, **H** Distribution of H3K9ac ChIP-seq peaks across genomic regions (**G**) and relative to TSS (**H**). **I** The left is differential genes related to glucose metabolism through ChIP-Seq, log2 fold change <0 represents the down-regulated genes, log2 fold change >0 represents the up-regulated genes. The right is Heatmap to show the expression levels of the selected genes through RNA-Seq (*P* < 0.05), Heatmap expression is the result of Z-score column-wise normalization. **J** H3K9ac distribution in the *Got1* gene showing differential H3K9ac peaks in E13.5 Control and *Sirt6* deficiency MEPM cells. **K**, **L** Enrichment of H3K9ac and SIRT6 at the *Got1* promoter in MEPM cells detected using the ChIP analysis, *n* = 4. **M** RT-qPCR analysis of *Got1* expression in E13.5 and E15.5 MEPM cells from Control and *Sirt6* cKO mice, *n* = 3. **N**, **O** Western blot analysis of GOT1 expression in E13.5 and E15.5 MEPM cells from Control and *Sirt6* cKO mice, *n* = 3. **P**, **Q** Representative immunohistochemistry of GOT1 in the palate of the Control and *Sirt6* cKO mice and quantitative analysis, Bar: 50 μm, *n* = 6. *p < 0.05, **p < 0.01, ***p < 0.001, ****p < 0.0001, ns not significant.

Considering that SIRT6 mainly plays a role through inhibiting H3K9ac, H3K18ac, and H3K56ac [21–23], we examined their acetylation level in the palate of *Sirt6* cKO mice. We found that H3K9ac increased much more significantly than H3K56ac and H3K18ac in E13.5 MEPM cells of the *Sirt6* cKO group (Fig. 5A, B), and the same changes were observed in E15.5 MEPM cells (Fig. 5C, D), E13.5 palate tissues, and E17.5 palate tissues (Fig. 5E, F). Then we conducted subsequent studies related to H3K9ac. To obtain insights into the genome-wide distribution of H3K9ac in MEPM cells and look for the target gene of *Sirt6* during palate development, we performed ChIP-seq experiments. We identified 3898 H3K9ac differential binding peaks, of which 2821 were up-regulated and 1077 down-regulated (Supplementary Fig. 3). Notably, around 27% of newly emerging H3K9ac peaks localized within promoter regions, and H3K9-enriched regions were mainly distributed around 0–1 kb up or downstream of the transcription start site (TSS) (Fig. 5G, H). To find the mechanism affecting glycolysis, we screened out the genes related to glucose metabolism that differ in both ChIP-seq and RNA-seq (Fig. 5I). Intriguingly, we found that the H3K9ac peaks of *Got1* were up-regulated in *Sirt6* loss MEPM cells (Fig. 5J). GOT1 is a key enzyme of the malate aspartate shuttle (MAS) and plays a crucial role in cell metabolic reprogramming [34–37]. ChIP-qPCR for H3K9ac also confirmed a significant increase of H3K9ac at the *Got1* gene in *Sirt6* loss MEPM cells compared to the Control (Fig. 5K). In addition, the *Got1* promoter region revealed significant enrichment with anti-SIRT6 ChIP (Fig. 5L) in MEPM cells. And both mRNA and protein expressions of *Got1* were higher in E13.5 and E15.5 *Sirt6* loss MEPM cells compared to the Control (Fig. 5M–O), confirming that SIRT6 repressed *Got1* gene expression. These results demonstrated that SIRT6 directly deacetylated H3K9 of the *Got1* gene, suppressing *Got1* expression. Moreover, immunohistochemical staining further confirmed GOT1 was mainly expressed in the mesenchyme and the expression of GOT1 in *Sirt6* cKO palate was increased (Fig. 5P, Q), which was consisted with *Sirt6* decrease. We hence hypothesized that *Got1* played a role in palatogenesis as the target of *Sirt6*.

### The H3K9 acetylation at the *Got1* gene is promoted by the acetylase TIP60 when *Sirt6* is knocked out

After SIRT6 was detected interacting with *Got1* and enhancing its H3K9 deacetylation levels (Fig. 5K, L), it suggested that histone acetyltransferase (HAT) might be involved. To determine which HAT was responsible for enhancing the acetylation level, we first observed the common acetyltransferase p300 (P300), but found that the expression of P300 decreased after *Sirt6* loss, indicating that P300 did not promote acetylation (Supplementary Fig. 4A, B). Moreover, as we detected an increase of lactate after *Sirt6* loss which could facilitate lactylation, also P300 was a writer of lactylation [38], we then observed whether there was an alteration in lactylation. The results indicated that *Sirt6* loss had no significant effect on lysine lactylation (Kla) level in MEPM cells (Supplementary Fig. 4A, B), and the effect of *Sirt6* loss on palate development was not through lactylation.

**Fig. 6:**
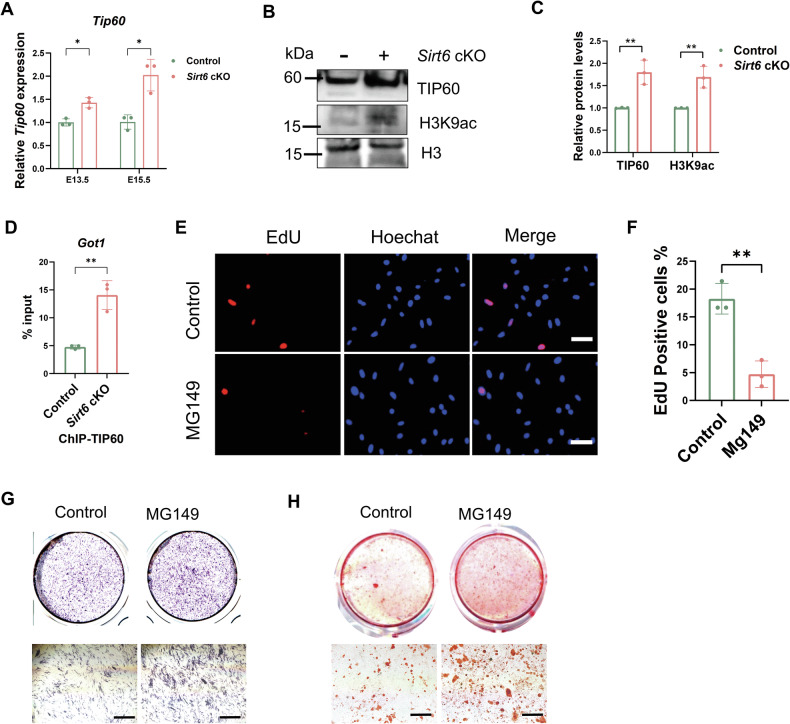
The H3K9 acetylation at the *Got1* gene is promoted by the acetylase TIP60 in *Sirt6* deficiency MEPM cells. **A** qRT-PCR analysis of *Tip60* expression in E13.5 and E15.5 MEPM cells, *n* = 3. **B**, **C** The levels of TIP60 and H3K9ac were measured by WB, *n* = 3. **D** Enrichment of TIP60 at the *Got1* promoter in MEPM cells detected using the ChIP analysis, *n* = 3. **E**, **F** MEPM cell proliferation was measured by an EdU staining assay, and MG149 was used at 10 μM in vitro, Bar: 50 μm, *n* = 3. **G**, **H** ALP (**G**) and Alizarin red (**H**) staining was used to observe the changes of osteogenic differentiation of MEPM cells at E15.5, Bar: 200 μm. *p < 0.05, **p < 0.01, ****p < 0.0001, ns not significant.

Therefore, we further detected the expression of other HATs by qRT-PCR and found that only tat-interacting protein 60 (*Tip60*) was stably expressed in *Sirt6* deficiency MEPM cells (Fig. 6A and Supplementary Fig. 4C–E). We also validated the increased expression of TIP60 in *Sirt6* deficiency MEPM cells by WB (Fig. 6B, C), indicating that TIP60 might play a role in acetylation in *Sirt6* deficiency MEPM cells. Further, it was showed that TIP60 was able to bind to the *Got1* gene, and the binding was increased in *Sirt6* deficiency MEPM cells (Fig. 6D). These results showed the balance of SIRT6 and TIP60 precisely regulated the expression of *Got1* through H3K9ac during palate development. To verify the role of TIP60 on *Got1* in MEPM cells, we added MG149 (TIP60 inhibitor) to MEPM cells and found that MG149 inhibited cell proliferation (Fig. 6E, F) and promoted osteogenic differentiation (Fig. 6G, H), which was the opposite with *Got1* activation on the cells and further confirmed the regulation of TIP60 to *Got1*.

### *Got1* inhibitor partially abrogates effects of *Sirt6* loss through GOT1-LDHA-TGFBR1/BMP2 signaling pathway

**Fig. 7:**
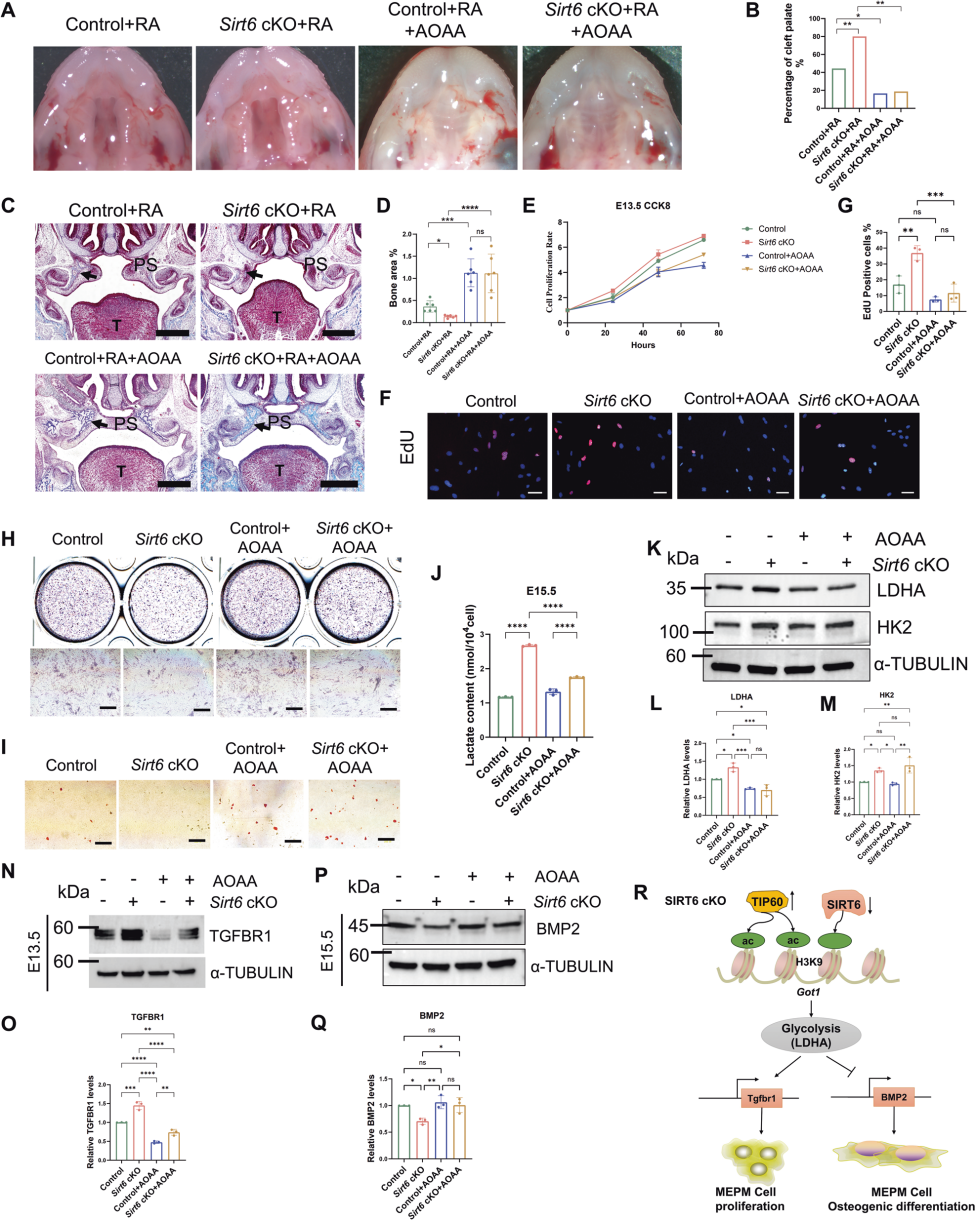
*Got1* inhibitor supplementation partially alleviates *Sirt6* loss-induced palatal dysplasia through the GOT1-LDHA-TGFBR1/BMP2 signaling pathway. **A** Macroscopic appearance of palates at E17.5. Pregnant mice at E10.5 were given a concentration of 50 mg/kg of RA by gavage, and AOAA was administered intraperitoneally at a dose of 5 mg/kg at E14.5 and E15.5. **B** Quantitative analysis of CP rate, *n* = 6 pregnant mice. **C**, **D** Masson trichrome staining of the maxillofacial region, Bar: 200 μm, *n* = 6, Note ossification of palate bone in Control and *Sirt6* cKO mice (black arrows). **E** The proliferation ability of E13.5 MEPM cells was detected by CCK-8, and AOAA was used at 200 μM in vitro. **F**, **G** E13.5 MEPM cell proliferation was measured by an EdU staining assay, Bar: 50 μm, *n* = 3. **H**, **I** ALP (**H**) and Alizarin red (**I**) staining was used to observe the changes in osteogenic differentiation of MEPM cells, Bar: 200 μm. **J** Quantitative analysis of lactate; **K**–**M** Western blot analysis of LDHA and HK2 expression in E15.5 MEPM cells from Control and *Sirt6* cKO mice. **N**–**Q** Western blot analysis of TGFBR1 (**N**, **O**) and BMP2 (**P**, **Q**) expression in E13.5 and E15.5 MEPM cells from Control and *Sirt6* cKO mice. **R** A model depicting the mechanism by which *Sirt6* cKO affects palate development. *Sirt6* cKO activates H3K9 acetylation by TIP60, affects its binding to the GOT1 promoter and glycolysis, and aggravates CP through the GOT1-LDHA-TGFBR1/BMP2 signaling pathway. *p < 0.05, **p < 0.01, ***p < 0.001, ****p < 0.0001, ns not significant.

To confirm the critical role of *Got1* in the effect of *Sirt6* on palate development, we administered aminooxyacetate (AOAA), the inhibitor of GOT1, to the *Sirt6* cKO mice. As the CP caused by *Sirt6* cKO only was not obvious, we combined it with RA (which is commonly used as an environmental factor inducing CP and could significantly increase the occurrence of CP in Fig. 1O, P) to investigate the role of GOT1 in the *Sirt6* cKO mice. Compared with the Control group, AOAA significantly mitigated the increased tendency of CP due to *Sirt6* cKO (18.8%, total of six pregnant mice with 16 *Sirt6* cKO fetuses collected, three with CP) (Fig. 7A, B and Supplementary Table 1). Then, it was observed that AOAA promoted the formation of fetal bone by Masson staining (Fig. 7C, D), which also coincided with in vitro experiments showing that AOAA inhibited the proliferation and promoted the weakening of osteogenic differentiation induced by *Sirt6* inhibition (Fig. 7E–I and Supplementary Fig. 5A, B).

To evaluate the role of *Got1* in glycolysis, we further suppressed the function of GOT1 by AOAA treatment in *Sirt6* loss MEPM cells. Our results found that AOAA attenuated the overexpression of LDHA in *Sirt6* loss MEPM cells, and inhibited lactate production. On the other hand, the effects of AOAA on HK2 between the control and control plus AOAA, *Sirt6* cKO and *Sirt6* cKO plus AOAA were not distinctive which meant it was LDHA but not HK2 was the major target of *Got1* (Fig. 7J–M). Furthermore, to clarify the relationship between GOT1 and TGFBR1/BMP2, we observed that the addition of AOAA inhibited the increase of TGFBR1 caused by *Sirt6* loss in early E13.5 MEPM cells (Fig. 7N, O), suggesting that *Sirt6* loss might affect TGFBR1 through GOT1-LDHA and thus affected early palatal process proliferation. Moreover, In E15.5 MEPM cells, AOAA was found to reactivate the decreasing BMP2 caused by *Sirt6* suppression (Fig. 7P, Q), suggesting that *Sirt6* loss might affect BMP2 through GOT1-LDHA and thus affect palatal process osteogenic differentiation. All of these findings suggest that *Sirt6* loss breaks the balance of H3K9 acetylation, which is then activated by TIP60, facilitates its binding to the GOT1 promoter, and aggravates CP through the GOT1-LDHA-TGFBR1/BMP2 signaling pathway (Fig. 7R).

## Discussion

Study has found that SIRT6 is decreased with preterm labor and regulates key terminal effector pathways of human labor in fetal membranes [39]. In addition, Wnt1-Cre;*Sirt6*^fl/+^ mice have shown decreased jawbone quality in a previous report, which shows the importance of *Sirt6* in craniofacial development [40]. In our study, the hypoplastic skeletal phenotype in CNCC derivatives-palatine bone, was noticeable at late embryonic stages and postnatal 12 weeks in *Sirt6* cKO mice, which was similar to previous studies that *Sirt6* deficiency inhibited craniofacial bone development [18, 40]. This implicated the prominent role of *Sirt6* in palatogenesis, thereby warranting further investigation. Interestingly, we also found that *Sirt6* loss promoted the proliferation of MEPM cells at early embryonic stages. This is in agreement with the latest finding that SIRT6 inhibits pulmonary arterial smooth muscle cell proliferation [41]. However, these findings indicated that *Sirt6* played a dual role in palate development. This is different from the previous literature, which mainly focused on the one-way effect of *Sirt6*. For example, *Sirt6*−/− mice show decreased chondrocyte proliferation and differentiation in tibial growth plates [42]. And *Sirt6* can promote the proliferation and differentiation of dental mesenchymal cells [43]. This may be attributable to the different effects of *Sirt6* at different developmental stages which cause a complicated superimposed effect [19, 44]. That might be also the reason there were no visible disorders in the *Sirt6* cKO mice craniofacial region. Previous studies mainly focused on proliferation and differentiation at the same time point, but we observed them at different developmental stages to better approximate the developmental characteristics at different periods. The dual role of *Sirt6* on palate development was also first found, as previous literature mainly focused on the one-way effect on palatogenesis, promoting or inhibiting the development of palate [45–48].

The dual effect made the effect of *Sirt6* on palate development complicated and induced insignificant CP. As RA, the metabolite of vitamin A, is a common inducer of CP by inhibiting the proliferation of palate cells [49], we used it as an environmental factor to mimic the etiology of CP. Surprisingly, we observed a significant increase of CP occurrence in fetal mice under *Sirt6* cKO-RA treatment, suggesting *Sirt6* had genetic susceptibility which could induce CP combining with environmental factors. This further confirmed the complexity of the etiology of CP.

*Sirt6* plays an important role in glucose metabolism [19]. Then, from the perspective of embryonic development and placental formation, glucose metabolism reprogramming is crucial for a successful pregnancy [50, 51]. Our previous study found that glycolysis enhanced in the early stages and then turned to OXPHOS in the late stages during palatogenesis [13]. So, we chose the influence of *Sirt6* loss on glucose metabolism during palatogenesis as a focus of the mechanistic study. We found that glucose metabolism reprogramming was disturbed with continuously activated glycolysis during palate development in *Sirt6* cKO mice, which might be the reason for the increased CP tendency caused by *Sirt6* inhibition. Our results are consistent with previous findings that SIRT6 overexpression in cardiomyocytes can coordinate metabolic remodeling from glycolysis to mitochondrial respiration during doxorubicin therapy [20].

To further investigate the mechanism underlying the sustained activation of glycolysis by *Sirt6* deficiency, we screened and observed that the H3K9 acetylation at the *Got1* gene was promoted, and *Got1* was consistently activated. In concordance with our findings, others have shown that *Got1,* as a key enzyme in MAS, can regenerate NAD^+^ to fuel glycolysis [35, 52]. During embryonic development, in placental tissue derived from “fast” blastocysts, expression of *Got1* is significantly higher compared to tissue from “slow” blastocysts [53]. Moreover, *Got1* is associated with the Sirtuins family, as *Sirt5* loss boosts tumorigenesis by raising the utilization of glutamine through GOT1 [54]. Our data also identified that *Got1* could activate glycolysis, and the inhibitor of *Got1* (AOAA) partially suppressed the increase of LDHA caused by *Sirt6* deficiency but didn’t affect HK2. This may be because NAD^+^ produced in *Got1*-mediated MAS can be used as feedstock for the pyruvate-to-lactate conversion process, which is catalyzed by LDHA rather than HK2 [52]. Significantly, AOAA could partially restrain the proliferative activity, abrogate the loss of osteogenic differentiation, and compensate for the CP caused by *Sirt6* cKO. These results are in agreement with the previous studies that inhibition of GOT1 in pancreatic cancer cells leads to cell death via ferroptosis [55] and can promote bone formation in the distal femur [56].

Epigenetic inheritance plays a vital role in embryonic development [57], and the activity of HATs and histone deacetylases is very critical for cellular homeostasis and cell fate [58–60]. Here, our results showed increased expression of the acetyltransferase TIP60 in *Sirt6*-deficient MEPM cells, and SIRT6 and TIP60 balanced the acetylation of *Got1*. So when *Sirt6* was knocked out, the acetylation balance of *Got1* was broken, causing the hyperacetylation of the *Got1* promoter, then abnormally activated glycolysis, ultimately resulting in a series of abnormalities that disturbed palate development.

Interestingly, protein lactylation has recently been proposed as a function of lactate, acting as a post-translational modification (PTM) of proteins to regulate gene expression [61]. In our study, lactate content was increased in *Sirt6* deficiency MEPM cells, but there was no difference in the expression of the lactylation. We speculated that this may be due to the competition between acetylation and lactation resulted in the change of lactylation was not obvious, while acetylation played a leading role. However, the specific mechanism remains to be further explored.

## Supplementary information

Supplementary Figure1

Supplementary Figure2

Supplementary Figure3

Supplementary Figure4

Supplementary Figure5

Supplementary materials and methods

Supplementary figure legends and tables

uncropped western images

## Data Availability

The ChIP-Seq and RNA-Seq raw data have been deposited to the National Center for Biotechnology Information (NCBI) under the BioProject number PRJNA1201795. The data supporting the findings of this study are available from the corresponding author upon reasonable request.
