# Supplementary figures and images for: Sirt6 loss activates Got1 and facilitates cleft palate through abnormal activating glycolysis
Source: Cell Death Dis. 2025 Mar 6;16(1):159. doi: 10.1038/s41419-025-07465-8 (PMC11885815; doi:10.1038/s41419-025-07465-8)

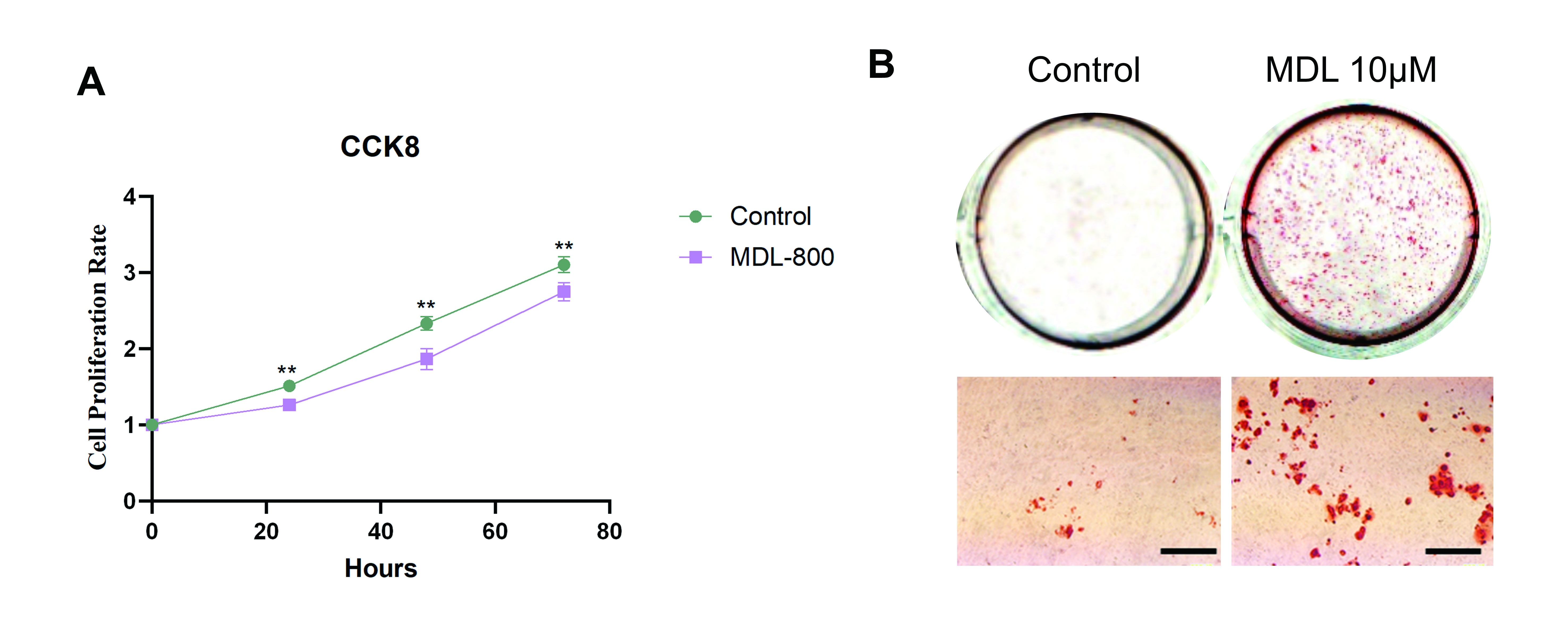

Supplement: Supplementary file 2 — Supplementary Figure2 [file 41419_2025_7465_MOESM2_ESM.tif]

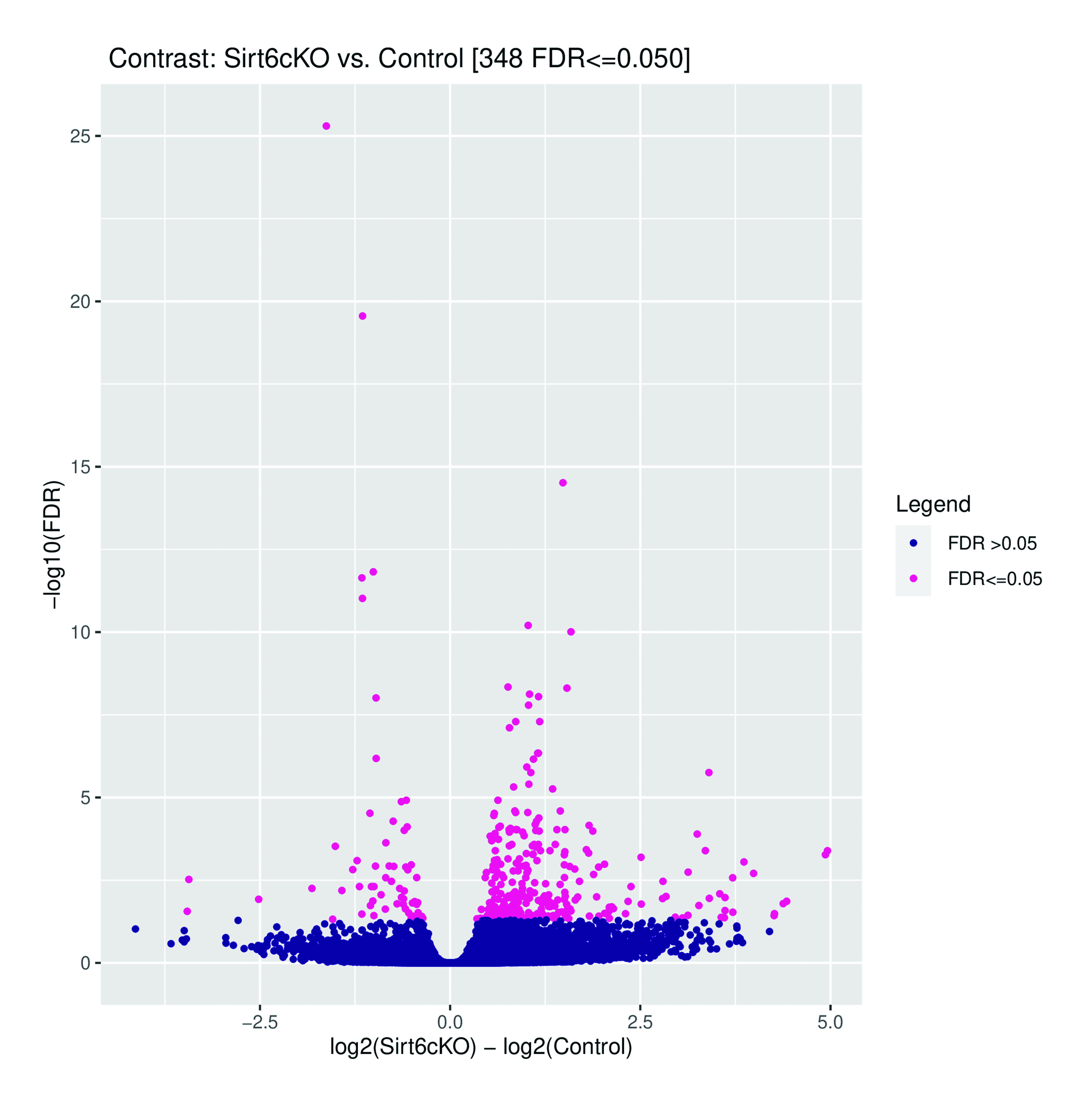

Supplement: Supplementary file 3 — Supplementary Figure3 [file 41419_2025_7465_MOESM3_ESM.tif]

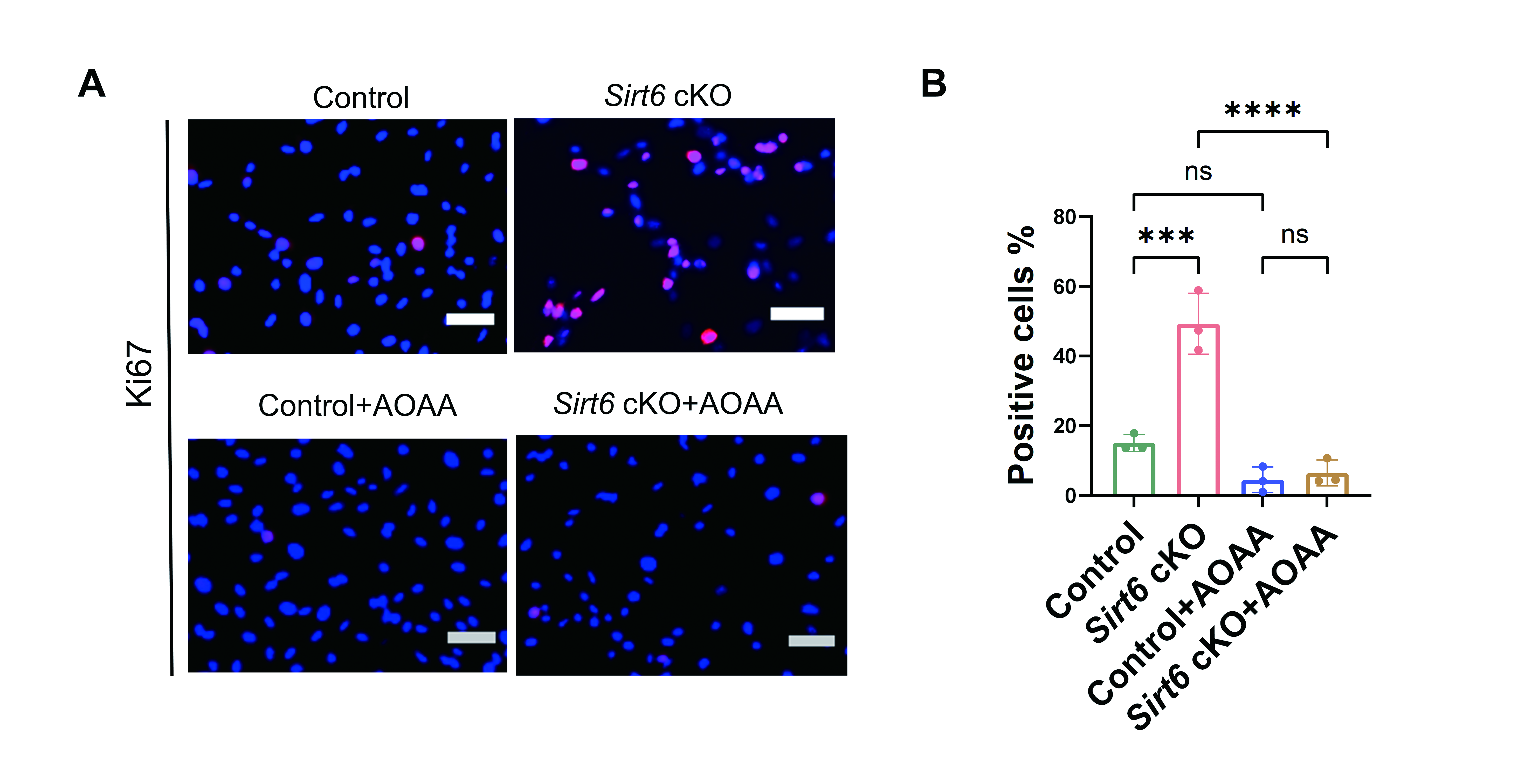

Supplement: Supplementary file 5 — Supplementary Figure5 [file 41419_2025_7465_MOESM5_ESM.tif]
