# Supplementary material for: Sirt6 loss activates Got1 and facilitates cleft palate through abnormal activating glycolysis
Source: Cell Death Dis. 2025 Mar 6;16(1):159. doi: 10.1038/s41419-025-07465-8 (PMC11885815; doi:10.1038/s41419-025-07465-8)
Supplement: Supplementary file 6 — Supplementary materials and methods [file 41419_2025_7465_MOESM6_ESM.docx]

***Sirt6* loss activates *Got1* and facilitates cleft palate through abnormal activating glycolysis**

**Supplementary Materials**

**Supplementary Material 1 Primer sequences used in this work**

| Name | Primers | Sequence(5'-->3') | Application |
| --- | --- | --- | --- |
| Wnt1-cre | wnt1-cre1 | GCTTGCATGATCTCCGGTAT | Genotyping |
|  | wnt1-cre2 | TGCCAGGATCAGGGTTTAAG | Genotyping |
| *Sirt6* loxp/loxp | Forward | AGTGAGGGGCTAATGGGAAC | Genotyping |
|  | Reverse | AACCCACCTCTCTCCCCTAA | Genotyping |
| *Got1* | Forward | AATCCCAGCCCTCCCATCTA | ChIP |
|  | Reverse | ATCCAGTCAGCCTTGGGTTC | ChIP |
| *Sirt6* | Forward | CTGAGAGACACCATTCTGGACT | RT-qPCR |
|  | Reverse | GGTTGCAGGTTGACAATGACC | RT-qPCR |
| *Runx2* | Forward | GATGACACTGCCACCTCTGA | RT-qPCR |
|  | Reverse | ATGAAATGCTTGGGAACTGC | RT-qPCR |
| *Opn* | Forward | CAAGGTAAGCCTGCAGTGGC | RT-qPCR |
|  | Reverse | ACTGCCAATCTCATGGTCGT | RT-qPCR |
| *Ocn* | Forward | GGGCAATAAGGTAGTGAACA | RT-qPCR |
|  | Reverse | GTCTTCAAGCCATACTGGTC | RT-qPCR |
| *Got1* | Forward | TCATCTGTGCGGTACGCTCC | RT-qPCR |
|  | Reverse | TTCACCGGCTTGAACCCCAA | RT-qPCR |
| *Tip60* | Forward | GCGGAGGTGGGGGAGAT | RT-qPCR |
|  | Reverse | AGCTTTCGGCCACTGATGTC | RT-qPCR |
| *Moz* | Forward | TTGACCCCCTAGACTGGCTT | RT-qPCR |
|  | Reverse | ACGCCTGGTTTTTCTGTCGT | RT-qPCR |
| *Morf* | Forward | AGTACCCCAGTGCTTTTCCG | RT-qPCR |
|  | Reverse | AGACAGGATGGGTGTCCACT | RT-qPCR |
| *Gcn5* | Forward | CAGATCCGCAAGGTCTACCC | RT-qPCR |
|  | Reverse | GCACTGGGGTGTGACTTGAT | RT-qPCR |

**Supplementary Material 2 Antibody used in this work**

| Antibody name | Catalog number | Manufacturer | Origin |
| --- | --- | --- | --- |
| SIRT6 | ab191385 | Abcam | UK |
| RUNX2 | 20700-1-AP | Proteintech | USA |
| OPN | A21084 | ABclonal | China |
| OCN | A6205 | ABclonal | China |
| αTUBULIN | 11224-1-AP | Proteintech | USA |
| Ki67 | 27309-1-AP | proteintech | USA |
| BMP2 | 66383-1-Ig | Proteintech | USA |
| LDHA | 19987-1-AP | Proteintech | USA |
| HK2 | 22029-1-AP | Proteintech | USA |
| H3K9ac | ab32129 | Abcam | UK |
| H3K18ac | A20735 | ABclonal | China |
| H3K56ac | A7256 | ABclonal | China |
| H3 | A22348 | ABclonal | China |
| GOT1 | 60317-1 | Proteintech | USA |
| Kla | PTM-1401 | PTM | China |
| p300 | ab275378 | Abcam | UK |
| TGFBR1 | ab235578 | Abcam | UK |
| TIP60 | 10827-1-AP | Proteintech | USA |
| Goat anti rabbit | SA00001-2 | Proteintech | USA |
| Goat anti mouse | SA00001-1 | Proteintech | USA |
| Goat anti rabbit | ZF-0317 | ZSGB-BIO | China |

**Supplementary Methods**

Animals

*Sirt6*^loxp/loxp^ mice were a gift from Sichuan University, and the Wnt1-Cre mice were presented by Dalian Medical University. *Sirt6*^loxp/loxp^ mice were interbred with Wnt1-Cre mice to obtain Wnt1-Cre/*Sirt6^loxp/loxp^* (*Sirt6* conditioned knockout, *Sirt6* cKO) mice and *Sirt6*^loxp/loxp^ (Control) littermates. As Wnt1-Cre/*Sirt6^loxp/loxp^* female mice were infertile, we crossed *Sirt6*^loxp/loxp^ female mice with the Wnt1-Cre/*Sirt6^loxp/loxp^* male mice for analysis of *Sirt6* cKO mutants and their littermates. We randomly selected mice in the *Sirt6* cKO group and the control group for the experiment, respectively. Primers for genotype identification were listed in Supplementary Material 1. All mice were approved by the Animal Care and Use Committee of the School of Stomatology, Capital Medical University (Beijing; License number: KQYY-202109-007, KQYY-202409-001), and all experiments complied with relevant regulatory standards.

Isolation of mouse embryonic palatal mesenchyme (MEPM) cells

*Sirt6*^loxp/loxp^ female mice and Wnt1-Cre/*Sirt6^loxp/loxp^* male mice were caged together, and the pregnant mice were euthanized at E13.5 and E15.5, respectively. Then we removed the embryos and took the MEPM cells for primary culture. MEPM cells were isolated from the palatal shelves and cultured in DMEM/F-12 medium (Cytiva, SH30023.01, China) with 5% serum (Vivacell, C04400-500, Shanghai, China) and 1% penicillin-streptomycin solution (Beyotime, C0222, Shanghai, China).

Histology and immunohistochemistry (IHC)

Firstly, we baked the paraffin sections at 65 °C for 1 hour, then deparaffinized and rehydrated them. Staining was carried out by immunohistochemical staining kit (ZSGB-BIO, ZLI-9001, Beijing, China). After antigen repair, the endogenous peroxidase blocker was added and incubated at room temperature for 10 minutes. The slices were then sealed in 10% goat serum (ZSGB-BIO, ZLI-9021, China) for 1 hour. The primary antibodies, BMP2 (1:200), TGFBR1 (1:150), H3K9ac (1:150), GOT1 (1:200), SIRT6 (1:150) were incubated overnight at 4°C. The secondary antibody was incubated for 1 hour at room temperature. The slices were stained with a standard DAB testing system (ZSGB-BIO, ZLI-9017, China) and counterstained with hematoxylin.

Micro-computed tomography (micro-CT) scanning

Heads of Control and *Sirt6* cKO mice were examined by radiography at 12 weeks old by micro-CT (Bruker, SKYSCAN1276, Germany). micro-CT reconstructions and quantitative analyses were accomplished using the SkyScan CT Analyzer (CTan) from regions of palate bone that spanned approximately 140 coronal slices. We used CTVox software to reconstruct 3D CT images.

Hematoxylin-eosin (HE) and Masson staining

HE staining: After we dewaxed the paraffin sections, they were stained with hematoxylin for 1 minute and soaked in 1% alcoholic hydrochloric acid solution for 8 seconds. Eosin was employed to stain the samples for 2 minutes. Then the slices were washed and dehydrated. Neutral resin sealed the slices. We applied a microscope to capture images.

For Masson staining, sections (5μm in thickness) were prepared, and a Masson Trichrome Staining Kit (Solarbio, G1346, Beijing, China) was applied according to the manufacturer’s instructions. We applied a microscope to capture images.

5-Ethynyl-20-deoxyuridine (EdU) staining

E12.5 pregnant mice were intraperitoneally administered at 50mg/kg EdU (Beyotime, C0075S, China). After 24 hours, the embryos were removed. Each section was added with 100μLClick reaction solution and incubated for 30 minutes away from light. Then each slice was added 1mL 1X Hoechst 33342 solution, incubated at room temperature without light for 10 minutes, and washed with phosphate-buffered saline (PBS) containing 3% bovine serum albumin (BSA).

For EdU assay in vitro, MEPM cells were seeded in the 12-well plate and incubated with EdU (10μm) for 2 hours the next day. Then the MEPM cells were washed with PBS containing 3% BSA for 3 minutes twice, before incubating with 4% Paraformaldehyde for 20 minutes. After soaking for 3 minutes twice, samples were permeated with 0.3% TritonX-100 in PBS, and incubated with click reaction solution (C0075S, Beyotime, China) at room temperature for 30 minutes away from light. Then we added Hoechst 33342 solution and incubated it at room temperature for 10 minutes away from light. At last, we used a microscope to capture images.

Immunofluorescence staining

The E13.5 MEPM cells were inoculated on 12-well plates. The next day, we discarded the medium, washed cells 3 times with PBS, then fixed cells with 4% paraformaldehyde for 20 minutes. After washing for 3 minutes twice, samples were permeated with 0.3% TritonX-100 in PBS. Next, MEPM cells were sealed at room temperature with 5% BSA for 30 minutes, and incubated overnight with primary antibodies at 4°C. On the next day, we added the diluent of the secondary antibody, incubated it for 1 hour, and then stained it with DAPI solution for 10 minutes. At last, we used a confocal laser microscope to capture images.

Skeleton stained with alizarin red and alcian blue

Embryos were taken at 17.5 days, fixed with 95% alcohol, and were defatted in acetone. After that, they were dyed with alizarin red and alcian blue. The samples were cleared in a series of diminishing concentrations of potassium hydroxide in glycerin. Then they were kept in glycerin. The cartilaginous skeletons were stained with blue, and the ossified skeletons were stained with red.

Chromatin immunoprecipitation (ChIP) assay

We performed the ChIP assays according to published protocols with some modifications (Beyotime, P2078, China). Briefly, MEPM Cells were suspended in 1% formaldehyde at 37℃ for 10  minutes and then blocked by glycine for 5  minutes. After centrifugation at 1,000g for 3  minutes, the precipitate was resuspended with 500 μl SDS lysis buffer and treated with ultrasound to cut genomic DNA. After centrifugation of the sample, the supernatant was added with protein A/G-agarose beads and rotated for 30 minutes at 4 ℃. Then we added the primary antibody to the supernatant and incubated them overnight at 4 ℃. Protein A/G-agarose beads were added and rotated at 4 ℃ for 1 hour. Finally, 200 μl freshly prepared elution buffer was added to the elute protein-DNA complex. NaCl was added and incubated at 65 °C for 4 hours to reverse crosslinking, then, DNA was purified for PCR analysis. Primers were exhibited in the Supplementary Material 1

ChIP-seq

The samples were fixed in 1% formaldehyde for Cross-Linking of DNA and Protein, then 0.125M glycine was added to block the crosslinking reaction. The chromatin was extracted from the sample by adding the Lysis Buffer. Then, samples were treated by ultrasound to fragment chromatin DNA. The sequencing libraries of input and IP were generated by NEBNext® UltraTM DNA Library Prep Kit for Illumina. Then, the library products corresponding to 100-500 bps were enriched, quantified, and finally sequenced on the Novaseq 6000 sequencer (Illumina) with the PE150 model. LC-Bio Technologies(Hangzhou)Co., Ltd completed the ChIP-Seq analysis.

RNA-Seq

The MEPM cells were collected, washed with PBS twice, and lysed with TRIzol reagent for the following RNA-seq in LC-Bio Technologies(Hangzhou)Co., Ltd. Then the quantity and purity of total RNA were controlled by NanoDrop ND-1000 (NanoDrop, Wilmington, DE, USA) and the integrity of RNA was detected by Bioanalyzer 2100 (Agilent, CA, USA). we used illumina NovaseqTM 6000 to double-end sequencing in PE150 mode according to standard procedures.

Seahorse XF glycolytic rate assay

The Seahorse XF24 extracellular flux analyzer (Seahorse Bioscience, MA, USA) was employed to analyze the extracellular acidification rate (ECAR) using the glycolytic rate test kit (Seahorse Bioscience, 103344-100, USA). Briefly, cells were plated at 2 × 10^4^ cells per well into 24-well Seahorse XF24 culture plates. The cells were incubated in Seahorse XF glycolysis rate assay solution containing glucose, glutamine, pyruvate, and HEPES buffer at 37 °C for 1 h in a CO_2_-free incubator. Then Rot/AA and 2DG were added into each well at the specified point.

Lactate production

We used a lactic acid assay kit (Abbkine, #ktb1100, Wuhan, China) to measure the lactate production according to the manufacturer's protocols. The cells were broken by ultrasound in an ice bath for 5 minutes. Then we added 50 µL of the cell supernatant to 50 µL of working reagent. Then we incubated them at 37 °C for 30 minutes. The standard curve was established. The production of lactate was calculated according to the number of cells.

Real-time quantitative reverse-transcription PCR (qRT-PCR)

Using Trizol (CWBIO, CW0580, Jiangsu, China) to extract total RNA, then it was reversed to cDNA using the HiScript Ⅲ RT SuperMix Perfect for qPCR kit (R333, Vazyme, Nanjing, China). The MagicSYBR mixture (CWBIO, CW3008H, China) was used to observe the expression of genes. They were run in triplicate. β-actin was used to normalize the final results. The comparative cycle threshold values (2−ΔΔCt) were calculated to analyze the results. Primers were showed in Supplementary Material 1.

Western blot analysis (WB)

We incubated the bands with primary antibodies overnight at 4 ° C, washed them with 1×TBST the next day, and incubated them with secondary antibodies for 1 hour at room temperature. The enhanced chemiluminescence reagent (MCE, K1005, New Jersey, USA) was used to develop the blots. And the band intensities were analyzed using the ImageJ software. The antibodies were listed in Supplementary Material 2.

Alizarin red staining (ARS)

After 28 days of osteogenic induction of E15.5 MEPM cells, we discarded the medium and washed the cells with PBS. 4% paraformaldehyde was used to fix the MEPM cells. Alizarin red staining solution (G8550, Solarbio, China) was used to stain the cells for 15 minutes. Then, we discarded the staining solution and washed the cells with PBS. At last, we used a microscope to capture images.

Alkaline phosphatase (ALP) staining

After 8 days of osteogenic induction of E15.5 MEPM cells, we discarded the medium and washed the cells with PBS. 4% paraformaldehyde was used to fix the MEPM cells. We used an alkaline phosphatase test kit (Beyotime, P0321M, China) to perform the ALP staining. Briefly, we formulated the staining solution according to the manufacturer's protocol. Then the MEPM cells were hatched with the staining solution for 40 minutes. Then, we discarded the staining solution and washed the cells with PBS. At last, we used a microscope to capture images.

Statistical analysis

All experiments were performed at least in triplicate *in vitro*, and all experiments were observed at least 6 samples *in vivo*. The number of biological replicates is stated in the figure legends as “*n*” and Supplementary tables. Calculations were done using the GraphPad Prism 10 software. Values were represented as mean ± SD. *T*-test were used to determine the statistical significance of differences between the two groups. One-way ANOVA with Tukey’s test was used to perform multiple comparisons. Fisher’s exact test was used to determine the statistical significance of the CP rate. *p* < 0.05 was considered statistically.
