# Supplementary material for: Sirt6 loss activates Got1 and facilitates cleft palate through abnormal activating glycolysis
Source: Cell Death Dis. 2025 Mar 6;16(1):159. doi: 10.1038/s41419-025-07465-8 (PMC11885815; doi:10.1038/s41419-025-07465-8)
Supplement: Supplementary file 7 — Supplementary figure legends and tables [file 41419_2025_7465_MOESM7_ESM.docx]

***Sirt6* loss activates *Got1* and facilitates cleft palate through abnormal activating glycolysis**

**Supplementary Fig. 1 Expression of SIRT6 in palate development and generation of *Sirt6* conditional mutant mice in Wnt1 lineages**

**A-C** The expression of SIRT6 in the palate of E12.5-18.5 was detected by qRT-PCR and WB, n=6. **D, E** Immunohistochemical analysis against of SIRT6, Bar: 40μm, n=6, PS: palatal shelves. **F** Genotyping for *Sirt6*^loxp/loxp^ and Wnt1-cre, Mut: Mutant (453 bp), Het: Heterozygote (399 bp and 453 bp). **G, H** SIRT6 protein levels in the palate of E14.5 Control and *Sirt6* cKO mice, n=6. **I, J** SIRT6 protein levels in E13.5 MEPM cells, n=3. **K, L** Immunohistochemistry of SIRT6 in E13.5 Control and *Sirt6* cKO mice, n=6. **M** Macroscopic appearance of palates at E17.5, pregnant mice at E10.5 were given a concentration of 50 mg/kg of RA by gavage, OSS_128167 was administered intraperitoneally at a dose of 10mg/kg at E10.5, E12.5 and E14.5. **N** Quantitative analysis of CP rate, n=6 pregnant mice. **p* < 0.05, ***p* < 0.01, ****p* < 0.001, *****p* < 0.0001, ns: not significant.

**Supplementary Fig. 2** **Effect of SIRT6 agonist MDL on proliferation and osteogenesis of MEPM cells**

**A** CCK-8 was used to detect the difference between Control and MDL-800 MEPM cells in E13.5, MDL-800 was used at 10μM. **B** Alizarin red S staining was used to observe the changes of osteogenic differentiation of Control and MDL-800 MEPM cells in E15.5, Bar: 200μm. ***p* < 0.01.

**Supplementary Fig. 3 The differentially enriched genes were detected in ChIP-Seq**

Differentially enriched gene volcano map, log2(Sirt6cKO)-log2(Control)<0 represents the down-regulated genes, log2(Sirt6cKO)-log2(Control)>0 represents the up-regulated genes, FDR<=0.5 represents a statistically significant difference.

**Supplementary Fig. 4 Expression of lysine lactylation and histone acetyltransferase (HAT) when *Sirt6* is knocked out**

**A, B** The levels of Kla, P300 were measured by WB, n=3. **C-E** qRT-PCR analysis of monocytic leukemia zinc finger protein (*Moz/Kat6a*), MOZ-related factor (*Morf*/*Kat6b*), general control non-repressible 5 (*Gcn5*/*Kat2a*) expression in E13.5 and E15.5 MEPM cells, n=3. *****p* < 0.0001, ns: not significant.

**Supplementary Fig. 5 GOT1 inhibitor partially attenuated the increased expression of Ki67 caused by SIRT6 deficiency**

**A, B** Immunofluorescence analysis of Ki67 in E13.5 MEPM cells, and AOAA was used at 200μM *in vitro*, Bar: 50μm, *n* = 3.

**Supplementary Table1 Frequency of cleft palate at 17.5**

| Group | Frequency of cleft palate at E17.5 |
| --- | --- |
| Control+RA | 44.4%(12/27) |
| *Sirt6* cKO+RA | 80.0%(16/20) |
| Control+RA+AOAA | 16.7%(2/12) |
| *Sirt6* cKO+RA+AOAA | 18.8%(3/16) |

**Supplementary Table2 Frequency of cleft palate at 17.5**

| Group | Frequency of cleft palate at E17.5 |
| --- | --- |
| PBS+RA | 37.5%(9/24) |
| *OSS+*RA | 73.5%(25/34) |
