# Supplementary material for: Sirt6 loss activates Got1 and facilitates cleft palate through abnormal activating glycolysis
Source: Cell Death Dis. 2025 Mar 6;16(1):159. doi: 10.1038/s41419-025-07465-8 (PMC11885815; doi:10.1038/s41419-025-07465-8)

Uncropped western images

Figure2

Fig. 2I

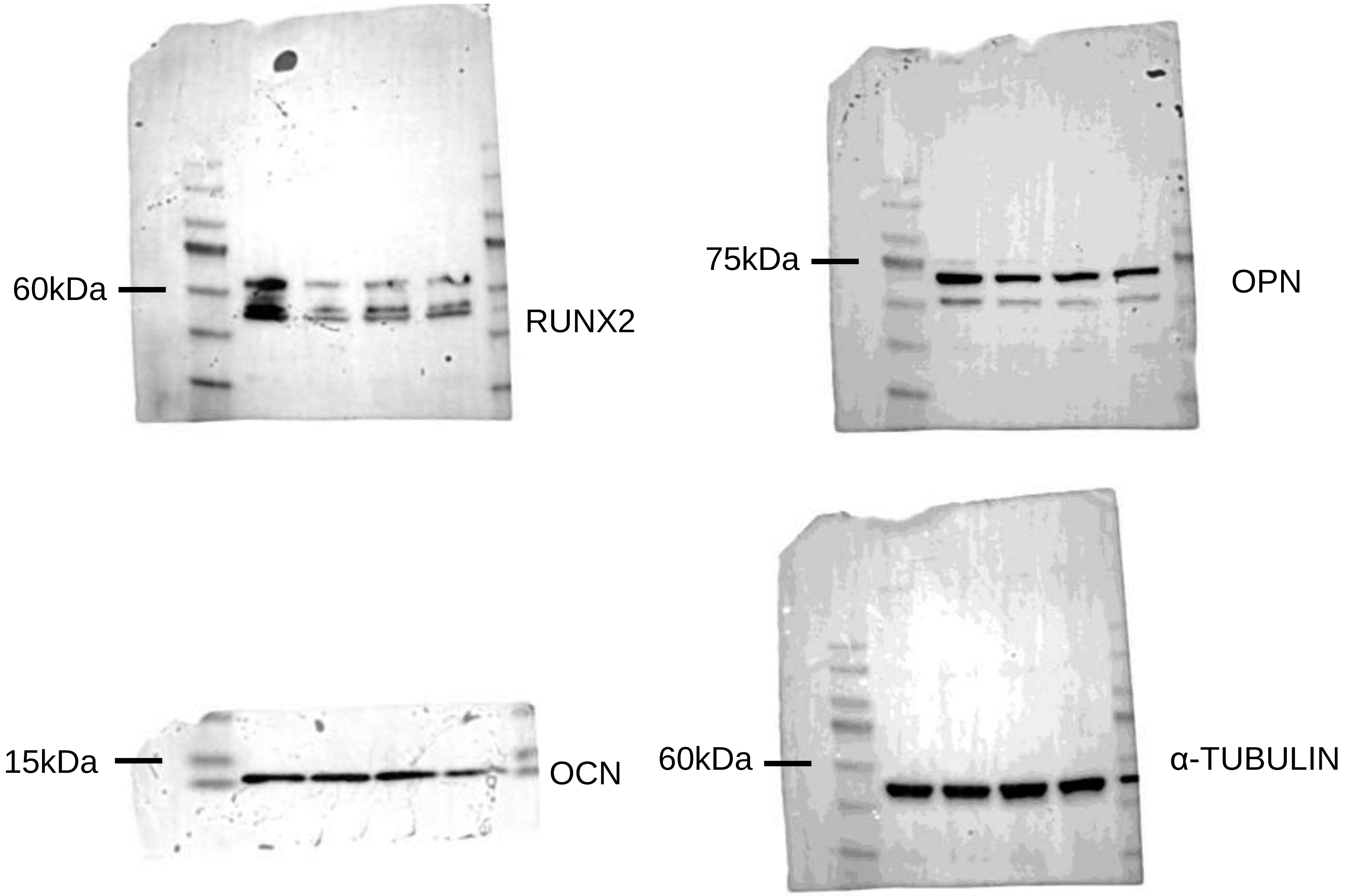

Figure3

Fig. 3C

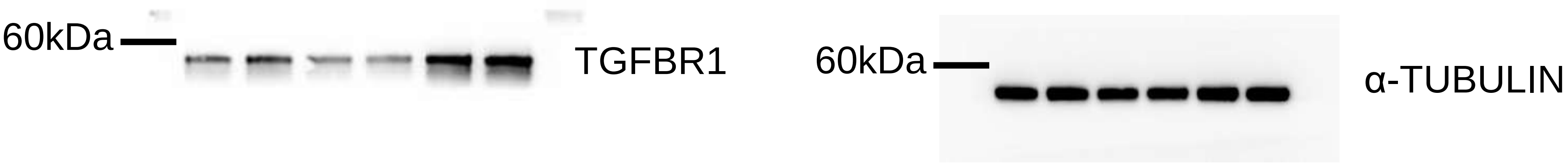

Fig. 3E

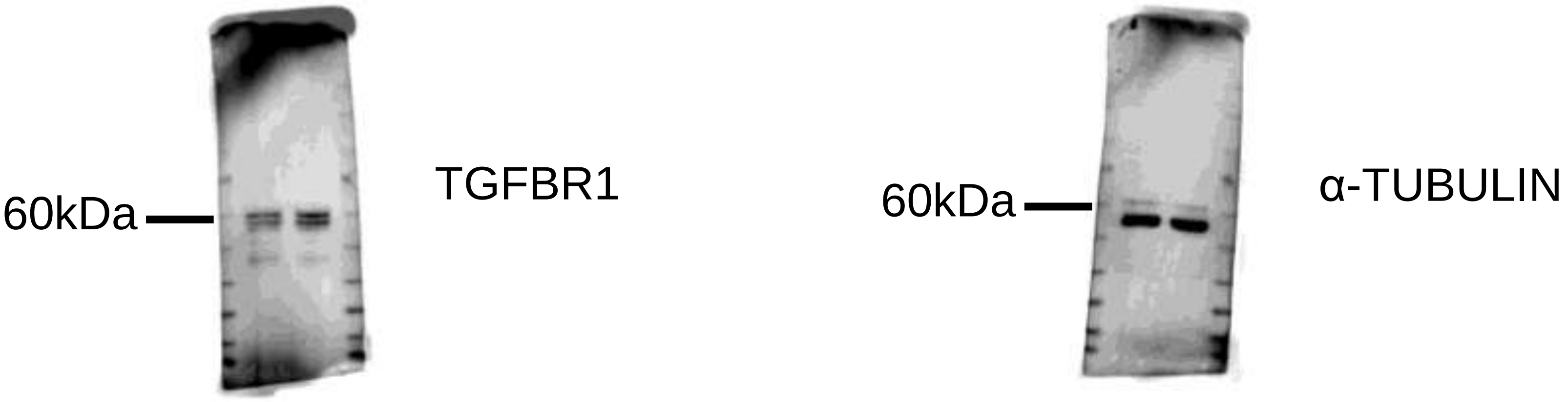

Fig. 3I

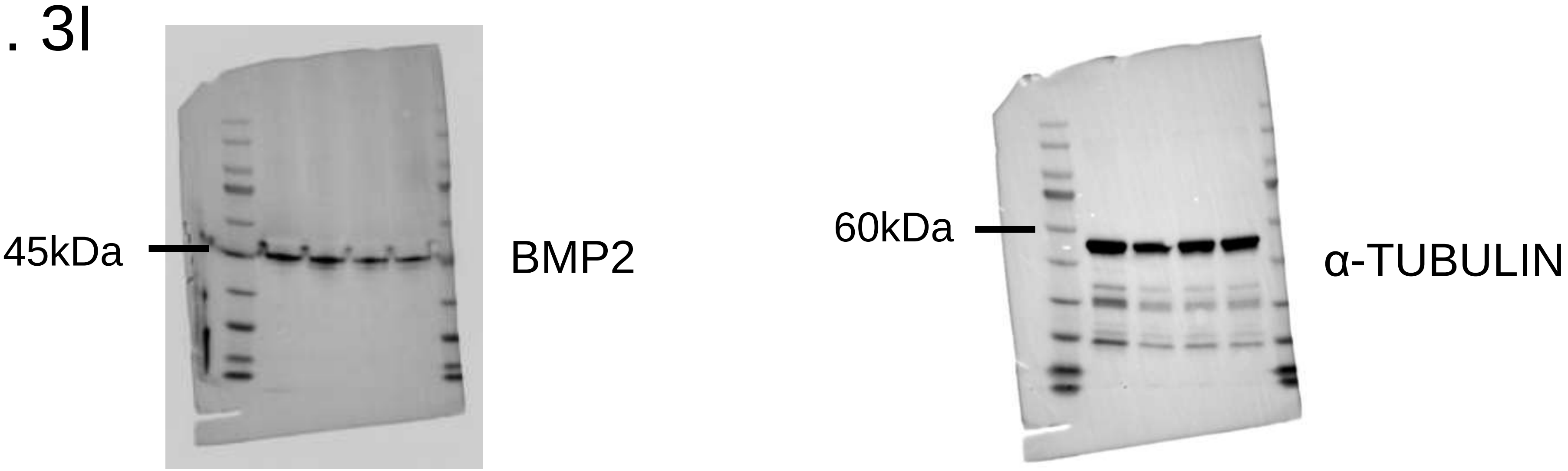

Fig. 3K

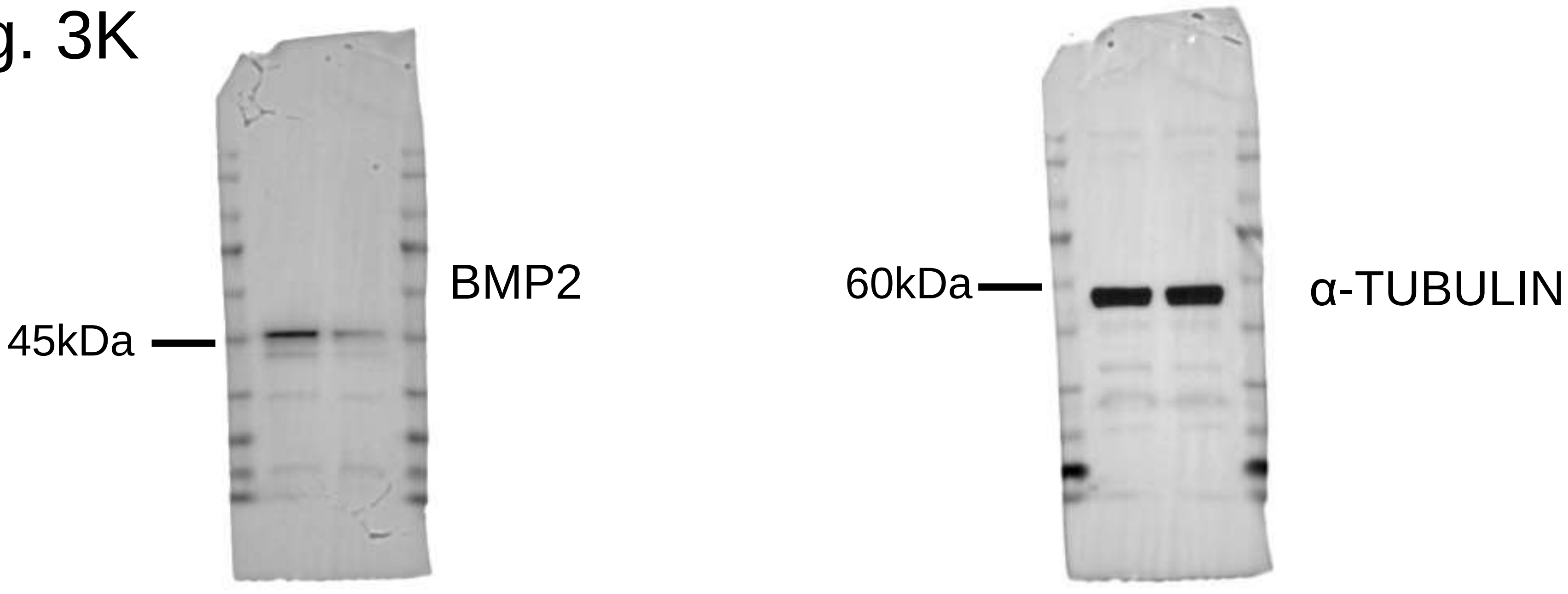

Figure4

Fig. 4E

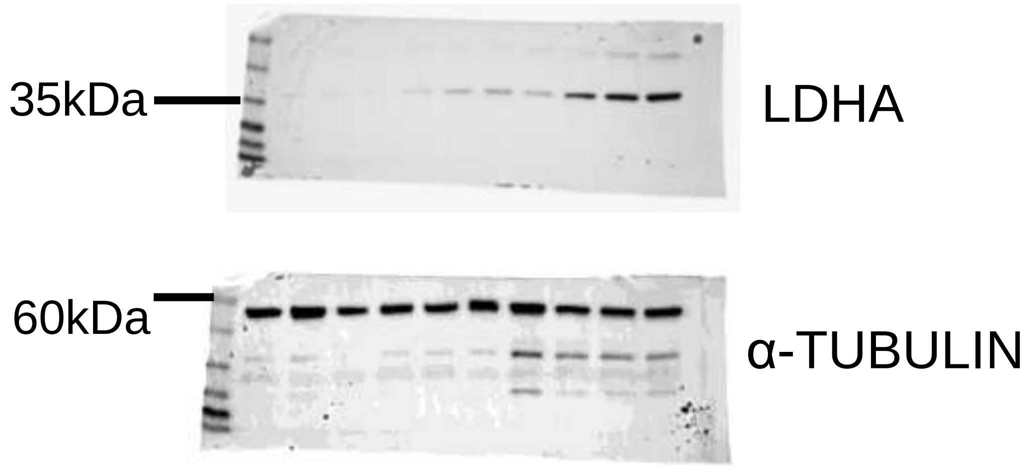

Fig. 4H

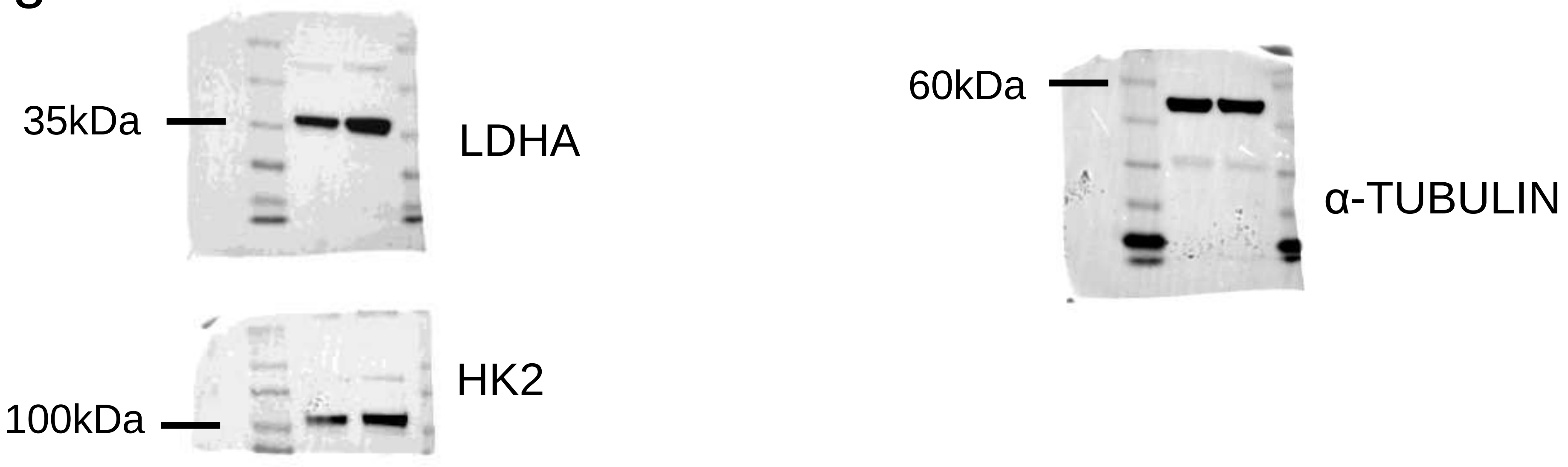

Fig. 4J

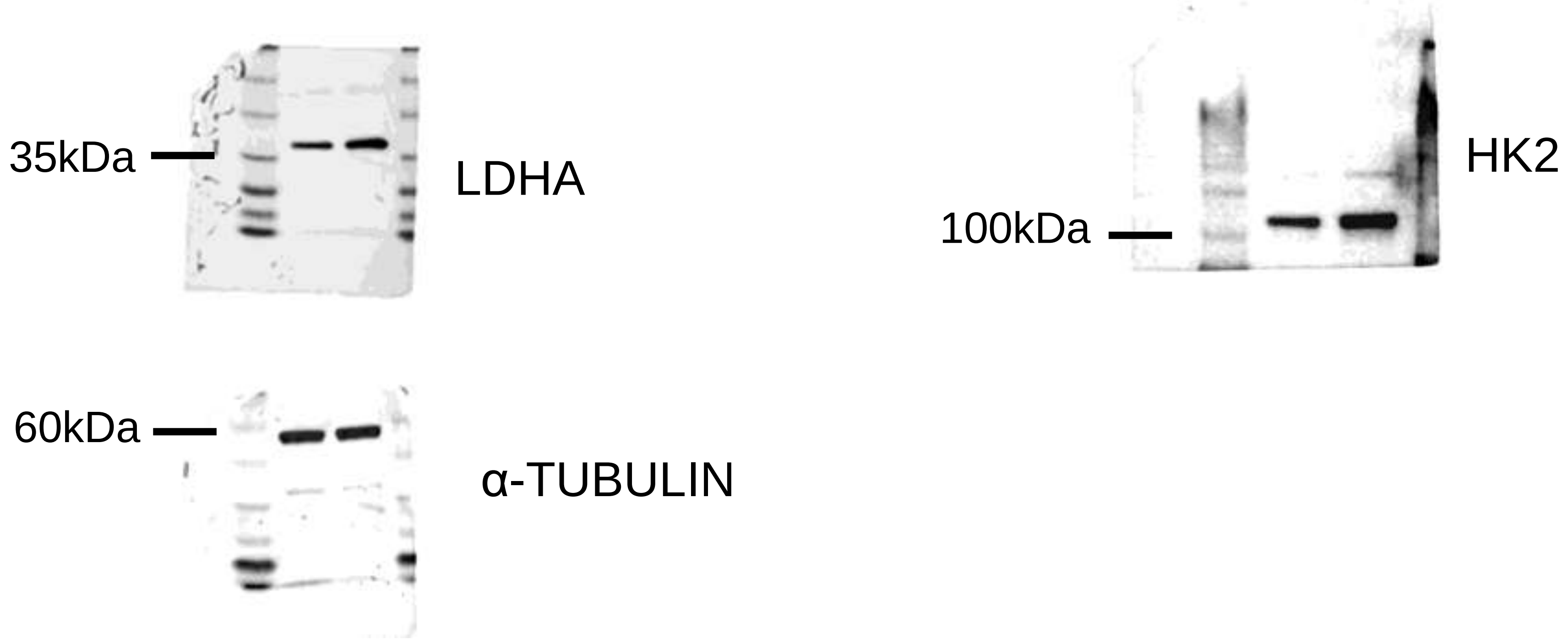

Figure5

Fig. 5A

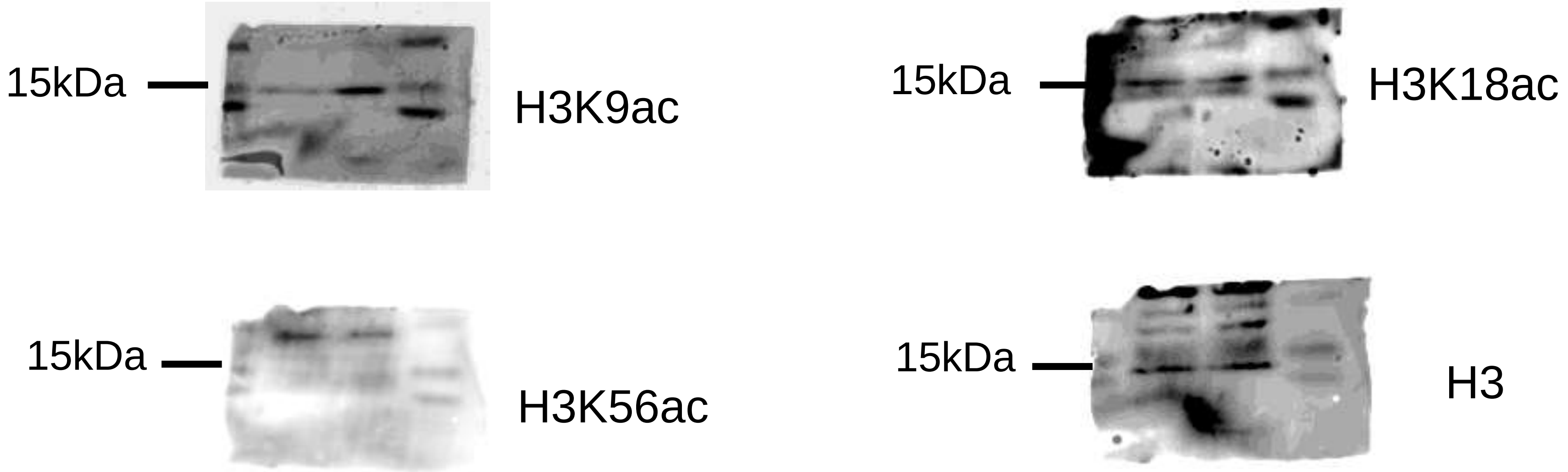

Fig. 5C

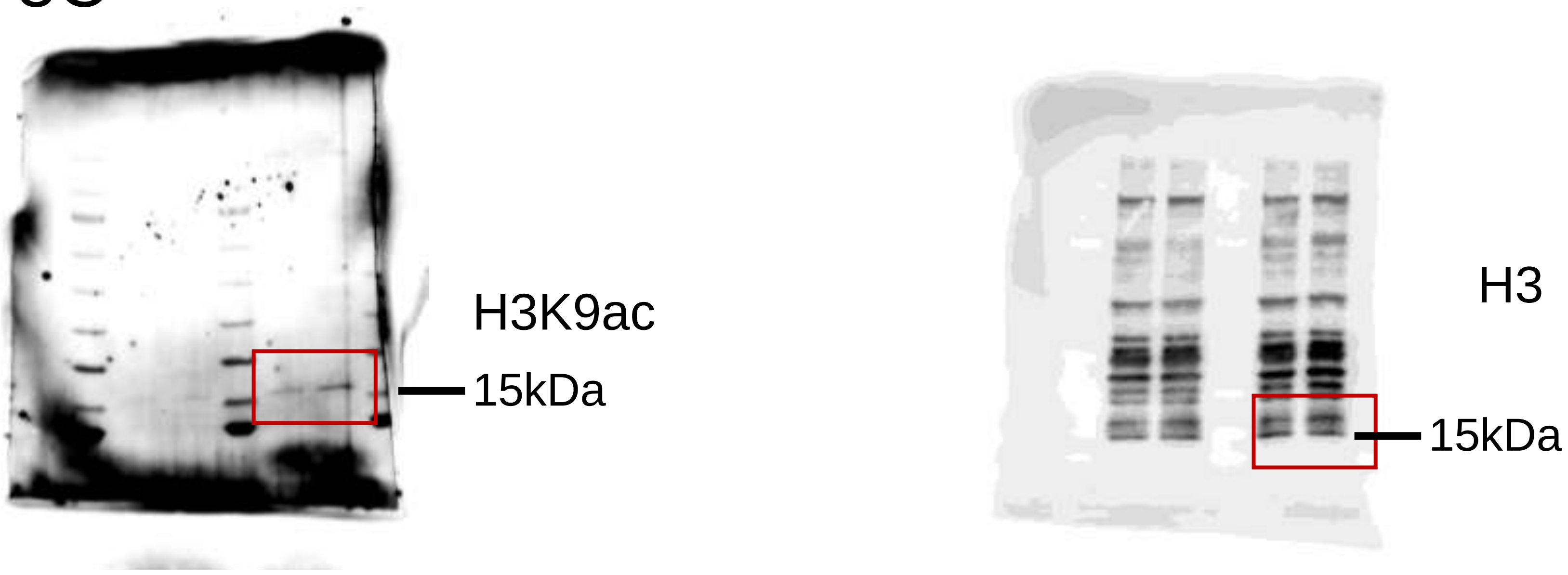

Fig. 5N

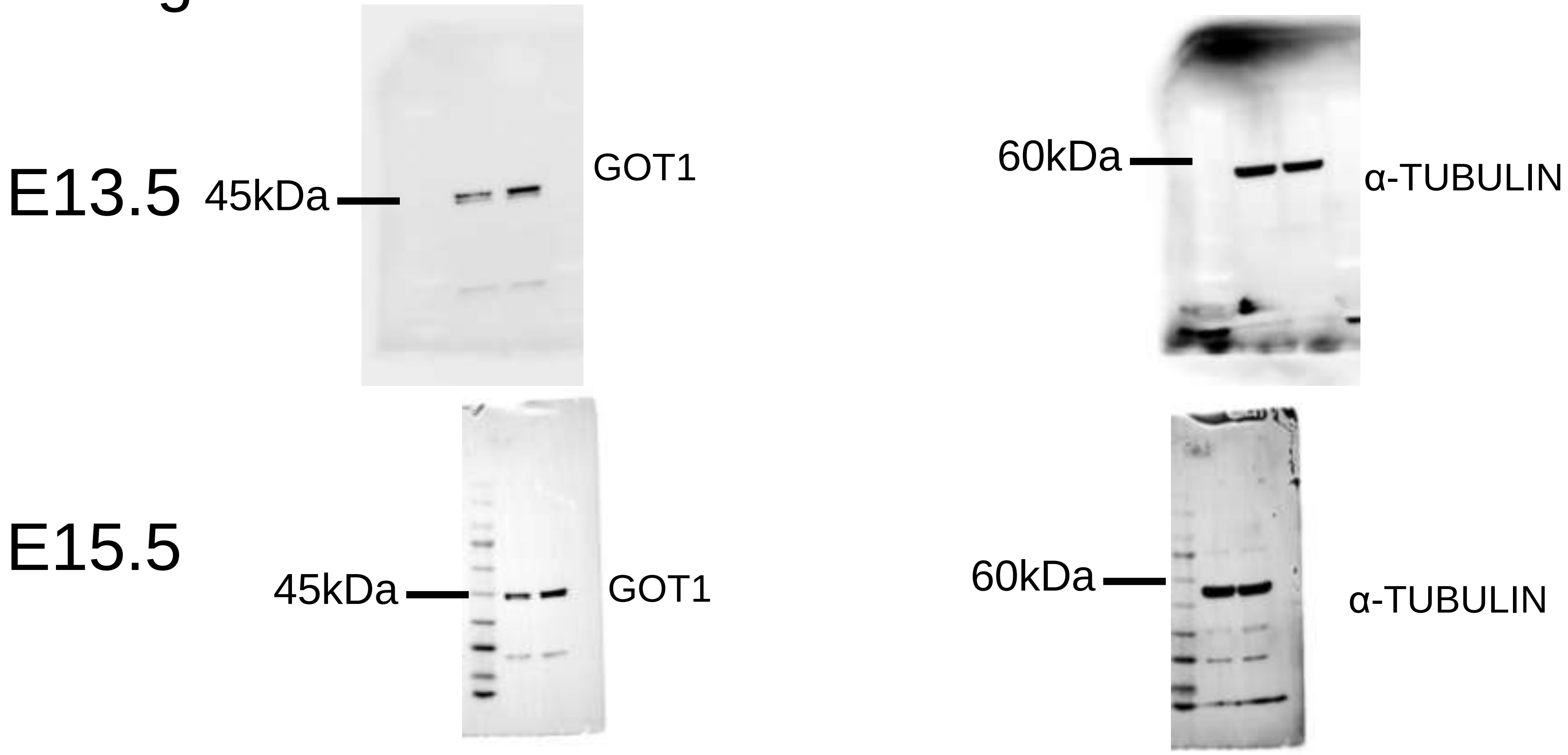

Figure6

Fig. 6B

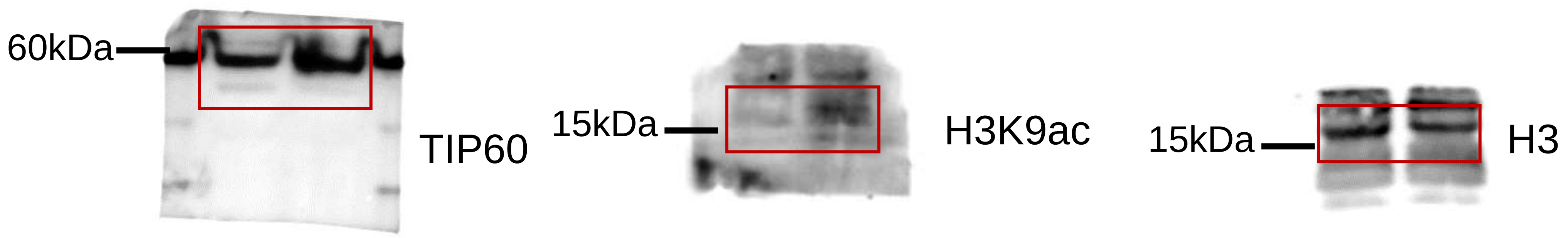

Figure7

Fig. 7K

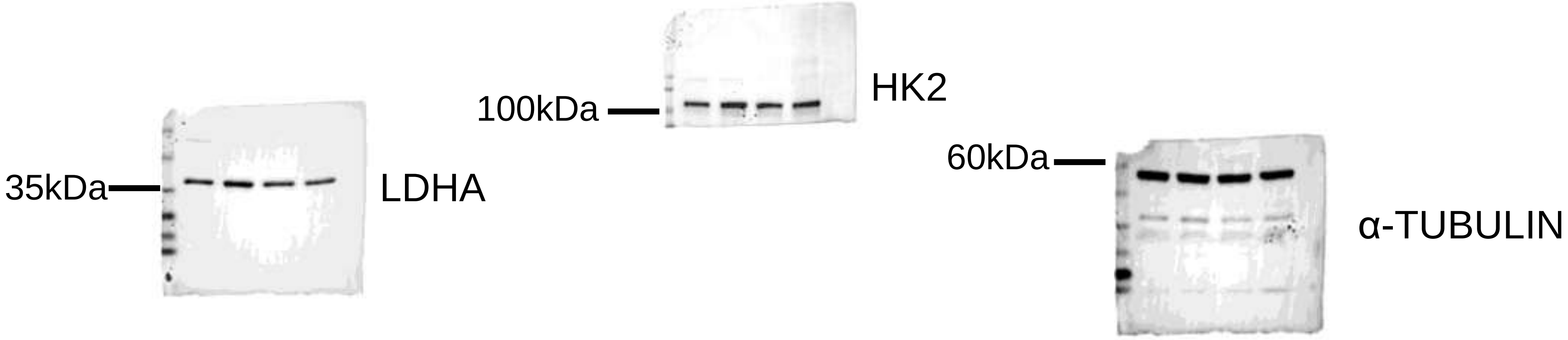

Fig. 7N

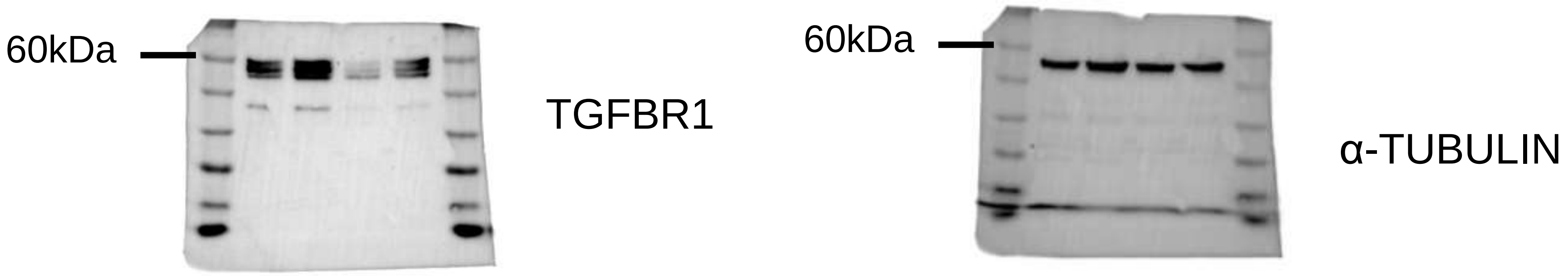

Fig. 7P

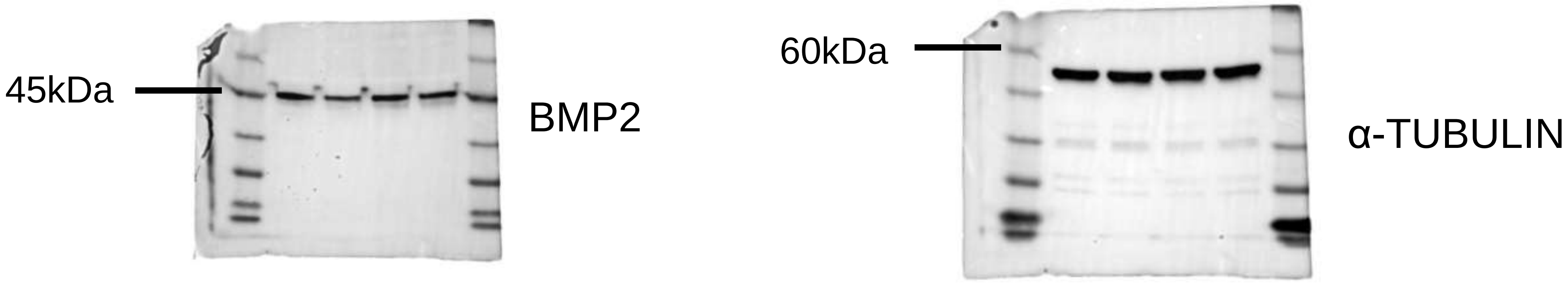

Supplementary Figure1

Supplementary Fig. 1B

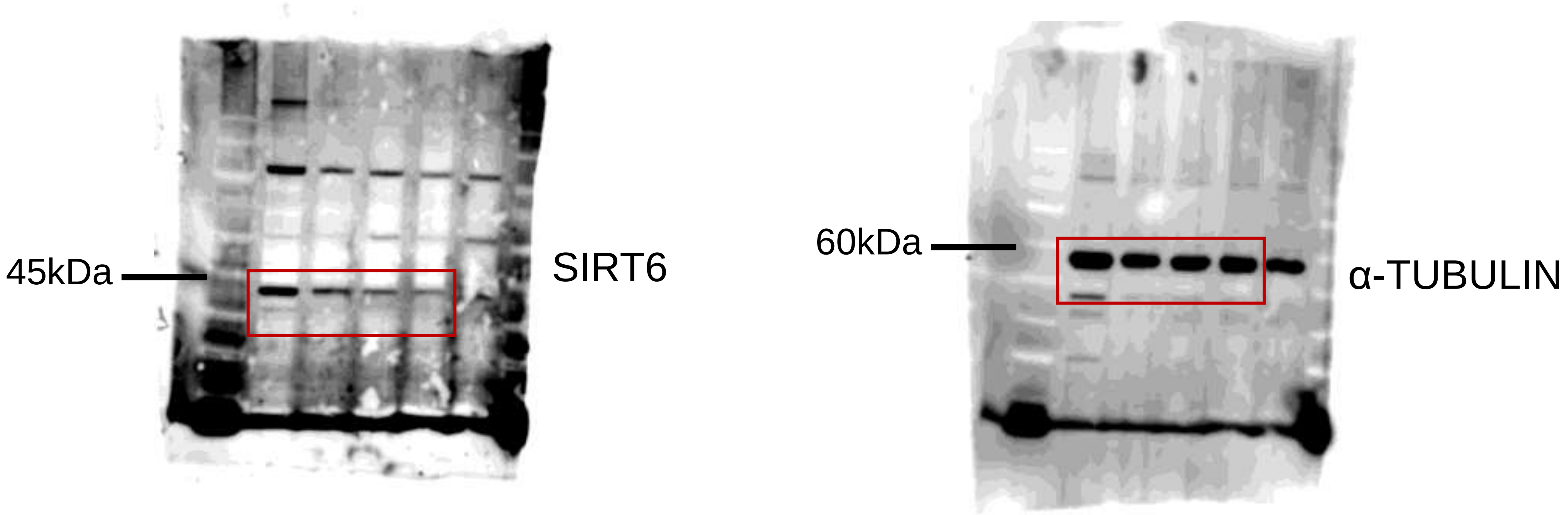

Supplementary Fig. 1G

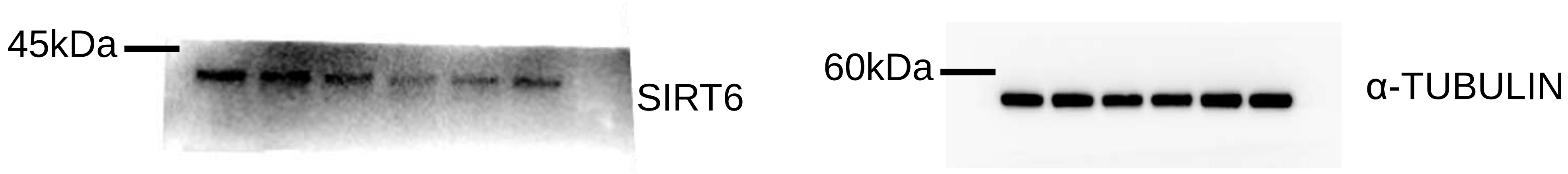

Supplementary Fig. 1I

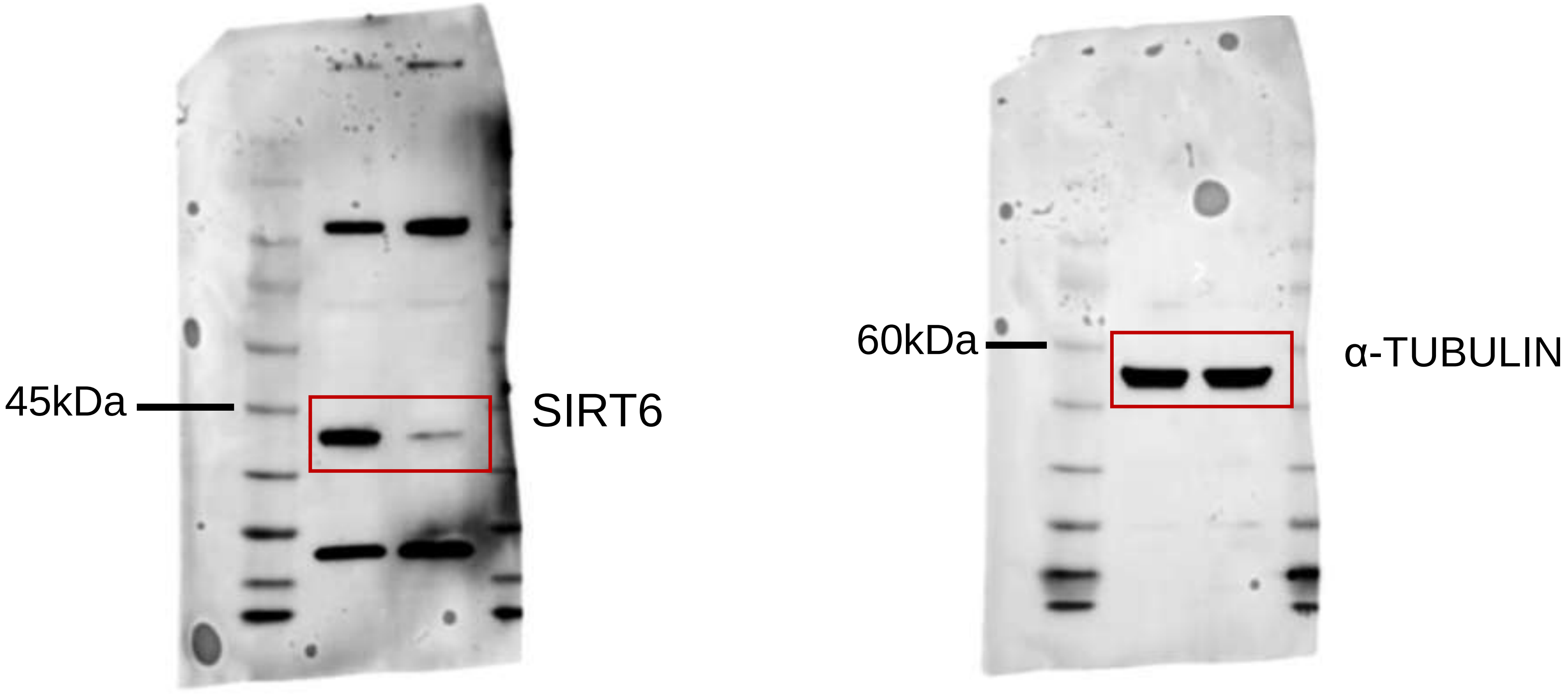

Supplementary Figure4

Supplementary Fig. 4A

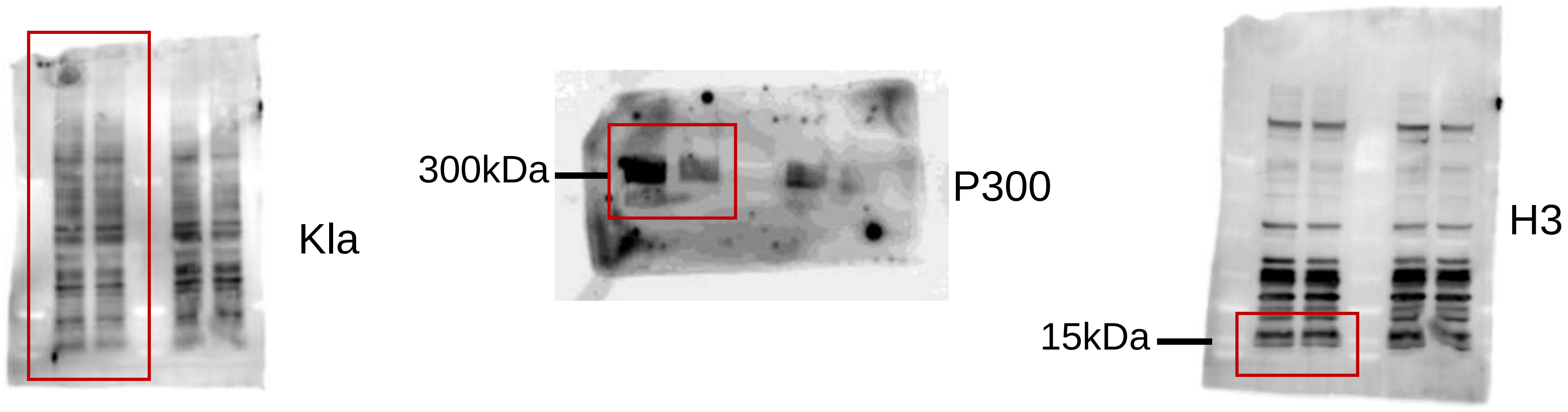

Supplement: Supplementary file 8 — uncropped western images [file 41419_2025_7465_MOESM8_ESM.pdf]
